# Dataset of NIR, MIR, FIR and NMR spectroscopy and GC-MS of real samples of e-liquids from Malaysia

**DOI:** 10.1016/j.dib.2025.111591

**Published:** 2025-04-30

**Authors:** Noor Hazfalinda Hamzah, Farah Natasha Mohd Aris, Nur Hayatna Mukhni, Stéphane Balayssac, Saïda Danoun, Véronique Gilard, Mohd Rashidi Abdull Manap

**Affiliations:** a Forensic Science Program, Faculty of Health Sciences, Universiti Kebangsaan Malaysia, 43000 Bangi, Selangor, Malaysia; b Department of Chemistry, Faculty of Science, Universiti Putra Malaysia, 43400, Serdang, Selangor, Malaysia; c Laboratoire Softmat, CNRS UMR 5623, Université de Toulouse, 31062 Toulouse, France; d Laboratoire SPCMIB, CNRS UMR 5068, Université de Toulouse, 31062 Toulouse, France; e Medical Microspectroscopy Research Group, Department of Experimental Medical Science, Lund University, 22180 Lund, Sweden

**Keywords:** ATR-FTIR, e-liquid, GC-MS, Malaysia, proton NMR

## Abstract

This dataset presents comprehensive spectroscopic and chromatographic profiling of 27 e-liquid samples including commercial formulations, a booster, and a nicotine solution (the e-liquids were collected in Ampang Jaya, Malaysia before April 2023). Fourier-transform infrared (FTIR) spectroscopy was performed across the near-, mid-, and far-infrared ranges (6000–80 cm^−1^), generating unique transmittance spectra for each sample. These spectra revealed vibrational bands characteristic of nicotine, propylene glycol, vegetable glycerine, and various additives, supporting rapid qualitative fingerprinting and comparison through OPUS software. ^1^H nuclear magnetic resonance (NMR) spectroscopy, conducted using a 600 MHz Bruker spectrometer with cryoprobe, enabled molecular-level identification of sample matrices. Signals from nicotine, propylene glycol, vegetable glycerine, and flavourings were resolved, with spectral expansion in the region of 5.5–10.5 ppm highlighting proton signals that differentiate nicotine forms and concentrations. Meanwhile, gas chromatography-mass spectrometry (GC-MS) analysis of all samples provided compound identification, detecting over 30 volatile compounds per sample including nicotine, esters, aldehydes, and nicotine-related degradation products. The results, available as chromatograms and tabulated peak profiles, highlight the presence of nicotine (including nicotine-N’-oxide), ethyl maltol, vanillin, and prohibited or potentially harmful compounds such as benzaldehyde derivatives. Collectively, these datasets offer a robust foundation for regulatory of nicotine in Malaysia, compositional fingerprinting, and substances screening of e-liquids using FTIR, GC-MS, and NMR as complementary tools.

Specifications Table

**Table:** 

Subject	Analytical Chemistry, Spectroscopy, Chromatography
Specific subject area	Chemical characterisation of e-liquids
Type of data	Raw and analysed data includes FTIR, NMR spectra, FID and GC-MS files
Data collection	FTIR spectra between 80 and 6000 cm^−1^ were acquired by a Bruker INVENIO-R (Universiti Putra Malaysia) spectrometer equipped with attenuated total reflection (ATR) (2mm) diamond with an accumulation of 64 scans at a spectral resolution of 4 cm^−1^ and processed by OPUS 8.7. Mid-infrared measurements were also carried out using an Bruker Alpha II spectrometer equipped with a platinum diamond ATR module, ZnSe beam splitter, RT-DLaTGS detector. The transmission spectra were recorded between 600 and 4000 cm^−1^ with a spectral resolution of 4 cm^−1^ and an accumulation of 32 scans. 1D NMR spectra were acquired on a Bruker Avance NEO spectrometer 600 MHz equipped with a 5 mm HPCN QCI cryoprobe. All NMR spectra were processed using TOPSPIN 4.0.8 software. GC-MS data were acquired by using Shimadzu Nexis GC-2030 gas chromatograph coupled with a GC-MS-QP2020 NX single quadrupole mass spectrometer, equipped with an AOC-20s auto-sampler and AOC-20i auto-injector. The obtained data were processed by LabSolutions GC-MS software.
Data source location	Institution: Department of Chemistry, Faculty of Science, Universiti Putra Malaysia Country: Malaysia. Latitude and longitude and GPS coordinates, for collected samples/data: 2°59′28.7"N 101°42′29.5"E Institution: SOFMAT Laboratory (BIBAC Team), Toulouse University Country: France Institution: Forensic Science Program, Faculty of Health Sciences, Universiti Kebangsaan Malaysia Country: Malaysia. 3°10′08.7"N 101°42′03.6"E
Data accessibility	FTIR data Repository name: Mendeley data Direct URL to data: Manap, Mohd Rashidi Abdull; Mukhni, Nur Hayatna; Hamzah, Noor Hazfalinda (2024), “The FIR MID NIR spectra of e liquids were acquired using a Bruker Invenio-R (Universiti Putra Malaysia) instrument equipped with an attenuated total internal reflection (ATR)”, Mendeley Data, V1 Data identification number: 10.17632/2cwtxdtstn.1 Direct URL to data: https://data.mendeley.com/datasets/2cwtxdtstn/1 Repository name: Manap, Mohd Rashidi Abdull; Mukhni, Nur Hayatna; Hamzah, Noor Hazfalinda (2025), “The MID spectra of e liquids were acquired using a Bruker Alpha II (Universiti Putra Malaysia) instrument equipped with an attenuated total internal reflection (ATR)”, Mendeley Data, V1
	Data identification number: 10.17632/ddrzk7t5nt.1 Direct URL to data: https://data.mendeley.com/datasets/ddrzk7t5nt/1 NMR data Repository name: Balayssac, Stéphane; Gilard, Véronique; Danoun, Saïda (2025), “The 1D 1H NMR spectra of e liquids”, Mendeley Data, V1, Data identification number: 10.17632/fc7xvdymgr.1 Direct URL to data: https://data.mendeley.com/datasets/fc7xvdymgr/1 GC-MS data Repository name: Aris, Farah Natasha; Hamzah, Noor Hazfalinda (2025), “The GC-MS Chromatogram of E-liquids”, Mendeley Data, V3. Data identification number: 10.17632/x9kh58w868.3 Direct URL to data: https://data.mendeley.com/datasets/x9kh58w868/3 Instructions for accessing these data: Click on the direct URL to obtain raw data.

## 1 Value of the Data

• The dataset integrates NIR, MIR, FIR, and ^1^H NMR spectroscopy with GC-MS analysis, providing a detailed chemical overview of real e-liquid samples. It enables qualitative and semi-quantitative detection of nicotine and additives, supporting comparative evaluation of analytical techniques for substance identification and profiling.
• This dataset can assist regulatory agencies and policymakers in assessing the reliability of different analytical methods for nicotine detection, facilitating more consistent labelling oversight, and supporting the development of regulatory standards for e-liquid products.
• Manufacturers and testing laboratories may use the data as a reference for selecting appropriate analytical tools in routine quality control, product authentication, and formulation consistency checks.

## 2 Background

This study involves examining the chemical composition of 27 samples, comprising 25 Malaysian commercial e-liquids, an e-liquid booster, and a nicotine solution reportedly used in the local e-liquid industry. These samples were collected before the exemption of nicotine-containing e-liquids from control under the Poisons Act 1952 by the Malaysian Ministry of Health on April 1, 2023. The authors specifically focus on assessing the presence of nicotine (both freebase and salt forms) in the e-liquid samples based on the New Zealand regulations [1]. The authors also diluted the samples during NMR analysis. However, under this condition, formal salt determination could not be performed using the NMR experimental setup. Nonetheless, this approach enabled the authors to explore the overall chemical composition of actual e-liquid samples. Full spectral and chromatogram data for each sample are accessible through Mendeley Data.

## 3 Data Description

The presented data includes both raw and analysed results from FTIR, NMR, and GC-MS experiments. Tables 1 and 2 provide the details of 25 e-liquid samples purchased from various vape shops in Ampang Jaya, Malaysia, along with an e-liquid booster and a nicotine solution (lot number: NIC_IPIN_MAY). All samples were labelled with a specific lot number: LV_542_MONTH OF PURCHASE_SAMPLE NUMBER. Information such as flavour, e-liquid category, labelling requirements, nicotine strength, liquid colour, smell, the presence of prohibited substances, and packaging details (in line with New Zealand requirements) is stated in the tables.

**Table 1:** Information on 27 samples analysed in this study.

No.	Lot number	Brand	Flavour	Category	Labelling									Nicotine Strength				Child warning sign	Liquid colour	Smell
					Safety of use instructions	Name and quantity of the substances	Volume or weight of the substance in the container	Manufacturing batch number	Manufacturer’s name and contact details	Expiry Date	PG %	VG %	Volume of samples (ml)	Nicotine strength as shown on the label (mg)	Nicotine form	Freebase nicotine not exceeding 20 mg/mL	Nicotine salt not exceeding 50 mg/mL			
1	LV_542_OKT_1	Cremano	Vanilla	2	Y	N	Y	N	Y	NA	35	65	70	NA	NA			Y	Brown	Vanilla
2	LV_542_OKT_2	Tickets Brew.co	Grape	1	Y	N	Y	N	Y	NA	30	70	60	3	NA			Y	Bright yellow	Grape
3	LV_542_OKT_3	Tickets Brew.co	Moji+bery	1	Y	N	Y	N	Y	NA	30	70	60	3	NA			Y	Yellow	Strawberry lime
4	LV_542_OKT_4	Peah Milf	Milky banabarb	2	Y	N	Y	N	N	NA	60	40	30	3	NA			Y	Amber	Banana milk
5	LV_542_OKT_5A	Ice King	Red ice	1	Y	Y	Y	N	Y	Y	50	50	50	3	NA			Y	Red	
6	LV_542_OKT_5B^1^	NA	NA	NA	NA	NA	NA	NA	NA	NA	NA	NA	NA	NA	NA			NA	Colourless	Watermelon
7	LV_542_OKT_6	Binjai	Fuji apple	1	Y	N	Y	N	Y	NA	40	60	60	3	NA			Y	Yellow	Fuji apple
8	LV_542_OKT_7	The Lunatics	Mempelam (Mango)	1	Y	N	Y	N	Y	NA	50	50	60	3	FB	Y		Y	Colourless	Mango
9	LV_542_OKT_8	Equal	Spritte	1	Y	N	Y	N	N	NA	50	50	60	NA	FB	NA		N	Pale yellow	Lime
10	LV_542_OKT_9	Equal	Reedbull	1	Y	N	Y	N	N	NA	50	50	60	3	FB	Y		N	Brown	Red Bull beverage
11	LV_542_OKT_10	Equal	Koka kola	1	Y	N	Y	N	N	NA	50	50	60	3	FB	Y		N	Pale brown	Coca Cola
12	LV_542_OKT_11	Mary Jane	Apple pear	1	Y	N	Y	N	Y	Y	50	50	30	3	FB (HTPC)	Y		N	Yellow	Apple pear
13	LV_542_OKT_12	Mary Jane	Butterscotch hazelnut coffee	2	Y	N	Y	N	Y	Y	50	50	30	NA	FB (HTPC)	NA		N	Amber	Butterscotch hazelnut coffee
14	LV_542_OKT_13	Premium Street Brew Tembakkau	Vanilla	2	Y	N	Y	N	Y	NA	HIGH	NA	30	NA	FB (HTPC)	NA		Y	Brown	Vanilla
15	LV_542_OKT_14	Mary Jane	Juicy grape	1	Y	N	Y	N	Y	Y	50	50	30	NA	FB (HTPC)	NA		N	Pale brown	Grape
16	LV_542_OKT_15	Tokyo	Blueberry	1	Y	N	Y	N	N	NA	60	40	30	NA	FB	NA		Y	Brown	Blueberry
17	LV_542_OKT_16	Challo Juice	Pineapple lychee	1	Y	N	Y	N	N	NA	50	50	30	18	FB (HTPC)	Y		Y	Yellow	Pineapple lychee
18	LV_542_OKT_17	Bangsawan Majapahit	Caramel coffee with butterscotch	2	Y	N	Y	N	Y	NA	50	50	30	22	FB (HTPC)	Y		Y	Brown	Caramel coffee butterscotch
19	LV_542_OKT_18	MeoMeoPeo	Grape	1	Y	N	Y	Y	Y	Y	60	40	10	35	NA			Y	Yellow	Grape
20	LV_542_OKT_19	Smart-D Brew	Blueberry crumble	2	Y	N	Y	N	Y	NA	50	50	10	35	NA			Y	Amber	Blueberry crumble
21	LV_542_OKT_20	57K District	Strawberry trifle	2	Y	N	Y	N	N	NA	60	40	10	35	NS		Y	Y	Yellow	Strawberry
22	LV_542_OKT_21	Sixty Symbols Hybrid	Caramel popcorn	2	Y	N	N	N	N	NA	NA	NA	10	35	NA			Y	Bright yellow	Caramel popcorn
23	LV_542_OKT_22	Dr-B	Mango crush	1	Y	N	Y	N	Y	NA	50	50	10	50	NA			Y	Pale yellow	Mango
24	LV_542_OKT_23	Equal	Geto rade	1	Y	N	Y	N	N	NA	50	50	10	50	NA			N	Pale yellow	Grape
25	LV_542_OKT_24	Geng Asap	Strawberry bacco	4	Y	N	Y	N	Y	NA	60	40	10	50	NA			Y	Bright yellow	Strawberry tobacco
26	LV_542_OKT_25	Hybrid Bangsawan	Hazelnut strawberry	2	N	N	Y	N	Y	NA	50	50	10	50	NS		Y	Y	Bright yellow	Hazelnut strawberry
27	NIC_IPIN_MAY	NA	No flavour	NA	NA	NA	NA	NA	NA	NA	NA	NA	NA	NA	NS			NA	Pale yellow	NA

*Note:* Category 1 relates to fruit, candy, alcohol, and beverage flavour; Category 2 relates to dessert, coffee, tea, and nuts flavour; Category 3 relates to menthol or mint flavour; Category 4 relates to spices, tobacco, and others. PG% is the percentage of propylene glycol present in the formulation. VG% is the percentage of vegetable glycerine present in the formulation. Nicotine strength is the number of milligrams (mg) of nicotine present in a formulation per bottle. 'NA' or empty cell refers to information unavailable; 'Y' refers to applicable; 'N' refers to not applicable. 'FB' or 'FB (HTPC – high throat hit pod compatible)' relates to freebase nicotine; 'NS' refers to nicotine salt.

1 LV_542_OKT_5B is an e-liquid booster with unknown compositions, packed together its e-liquid product LV_542_OKT_5A.

**Table 2:** Further details on 27 samples analysed in this study, as an extension of Table 1.

No.	Lot number	Prohibited substances																	Container			
		Long-chain parabens	Isothiazolinone	Phenoxyethanol	Triclosan	Sugars and sweeteners	Formaldehyde releasers	Probiotics	Polyethylene glycol	Diethylene glycol	Ethylene glycol	Glucuronolactone	Taurine	Colouring	Pharmacologically active substance	Vegetable oils other than glycerine	Caffeine	Mineral oils	Food grade plastics	Protected against breakage and leakage	Must have child-resistant closures	Anti-spill flow inserts
1	LV_542_OKT_1					Y													Y	N	Y	Y
2	LV_542_OKT_2					Y													Y	N	Y	Y
3	LV_542_OKT_3					Y													Y	N	Y	Y
4	LV_542_OKT_4													N					Glass (Y)	N	Y	Y
5	LV_542_OKT_5A																		Glass (Y)	N	Y	Y
6	LV_542_OKT_5B																		Glass (Y)	N	Y	Y
7	LV_542_OKT_6																		Y	N	Y	Y
8	LV_542_OKT_7																		Y	N	Y	Y
9	LV_542_OKT_8					Y								N					Y	N	Y	Y
10	LV_542_OKT_9					Y								N					Y	N	Y	Y
11	LV_542_OKT_10					Y								N					Y	N	Y	Y
12	LV_542_OKT_11																		Y	N	Y	Y
13	LV_542_OKT_12																		Y	N	Y	Y
14	LV_542_OKT_13					Y													Y	N	Y	Y
15	LV_542_OKT_14																		Y	N	Y	Y
16	LV_542_OKT_15																		Y	N	Y	Y
17	LV_542_OKT_16																		Y	N	Y	Y
18	LV_542_OKT_17																		Y	N	Y	Y
19	LV_542_OKT_18					Y													NA	N	Y	Y
20	LV_542_OKT_19					Y													Y	N	Y	Y
21	LV_542_OKT_20																		Y	N	Y	Y
22	LV_542_OKT_21																		Y	N	Y	Y
23	LV_542_OKT_22					Y													Y	N	Y	Y
24	LV_542_OKT_23					Y													Y	N	Y	Y
25	LV_542_OKT_24																		Y	N	Y	Y
26	LV_542_OKT_25																		Y	N	Y	Y
27	NIC_IPIN_MAY																		NA	NA	NA	NA

*Note:* Information on the inclusion of prohibited substances was recorded based on the ingredient list of each sample. 'NA' or empty cells indicate unavailable information. 'Y' denotes that the substance or feature is present in the formulation or packaging, while 'N' denotes that the substance or feature is absent from the formulation or packaging.

The transmittance spectra (in percentage transmittance, %T) were acquired for each sample between 80 and 6000 cm^−1^ covering near-infrared (NIR), mid-infrared (MIR), and far-infrared (FIR) spectra region. The e-liquid sample spectra are shown between Fig. 1, Fig. 2, Fig. 3, Fig. 4, Fig. 5, Fig. 6, Fig. 7, Fig. 8, Fig. 9, Fig. 10, Fig. 11, Fig. 12, Fig. 13, Fig. 14, Fig. 15, Fig. 16, Fig. 17, Fig. 18, Fig. 19, Fig. 20, Fig. 21, Fig. 22, Fig. 23, Fig. 24, Fig. 25, Fig. 26-26. Meanwhile, the nicotine solution spectrum is shown in Fig. 27.

**Fig. 1:**
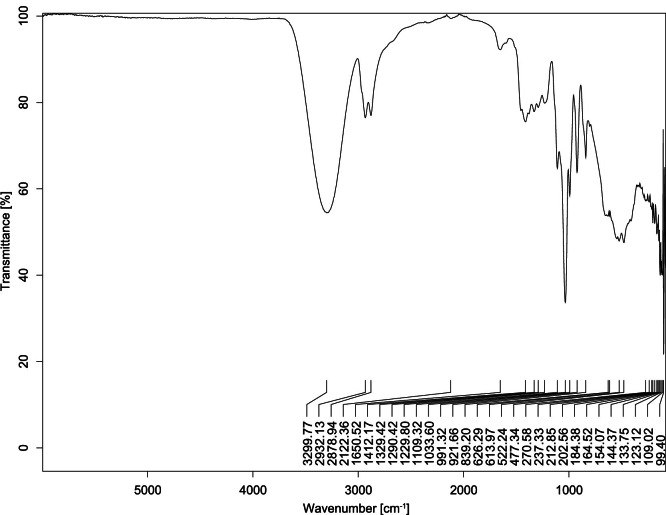
FTIR for LV_542_OKT_1.

**Fig. 2:**
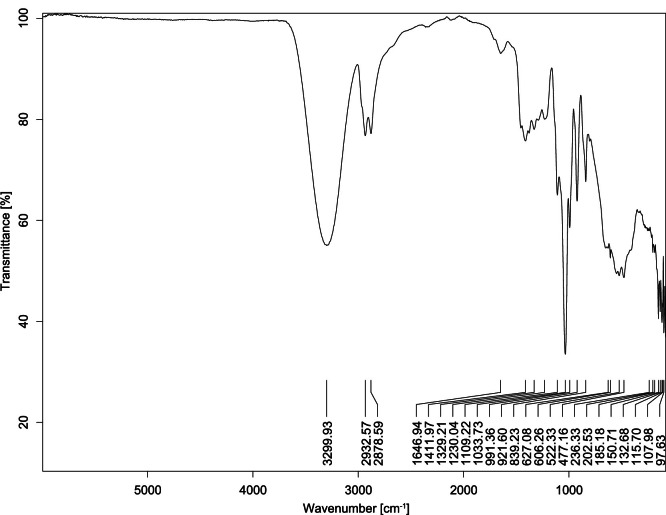
FTIR for LV_542_OKT_2.

**Fig. 3:**
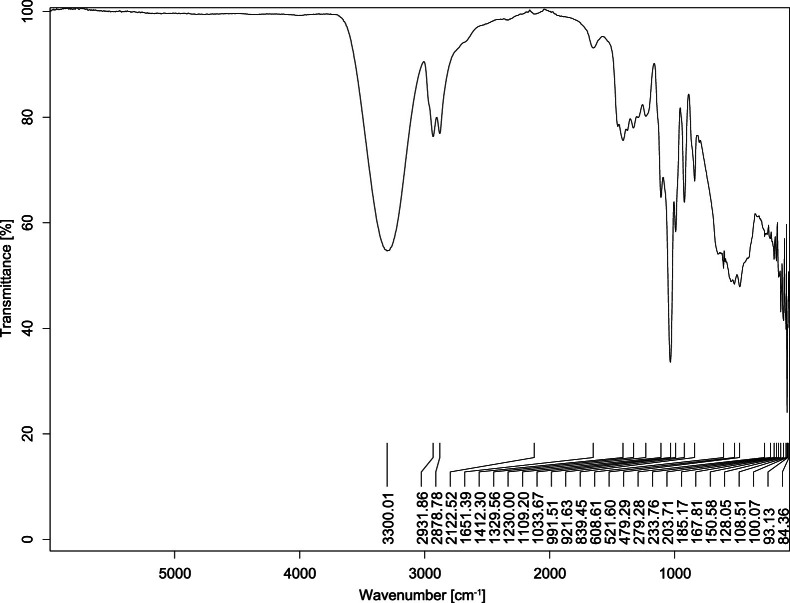
FTIR for LV_542_OKT_3.

**Fig. 4:**
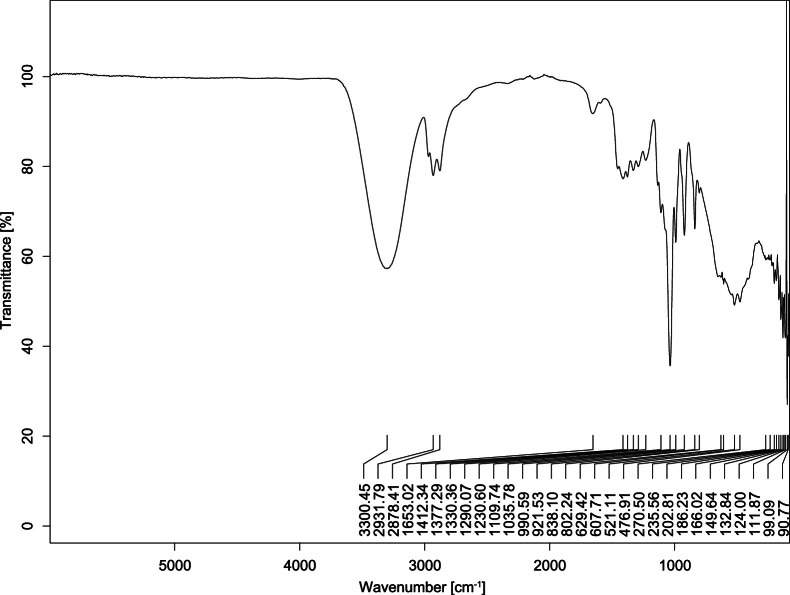
FTIR for LV_542_OKT_4.

**Fig. 5:**
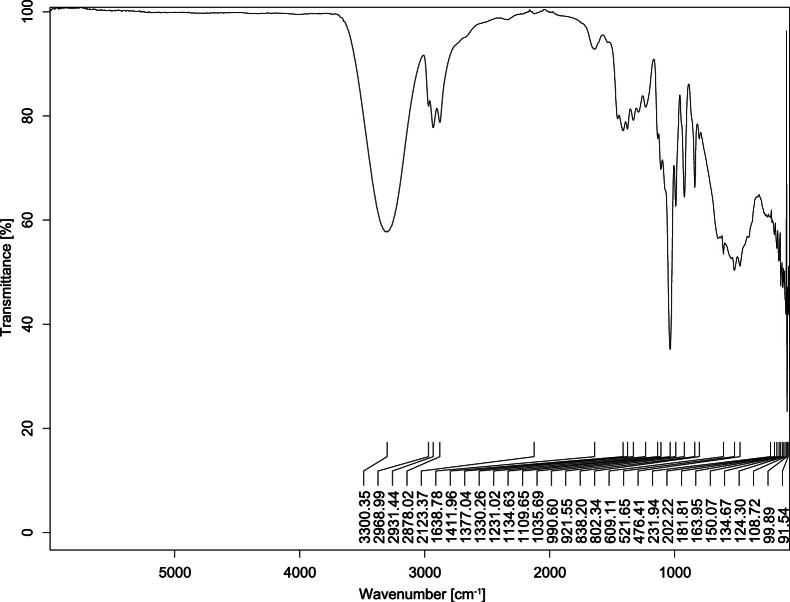
FTIR for LV_542_OKT_5A.

**Fig. 6:**
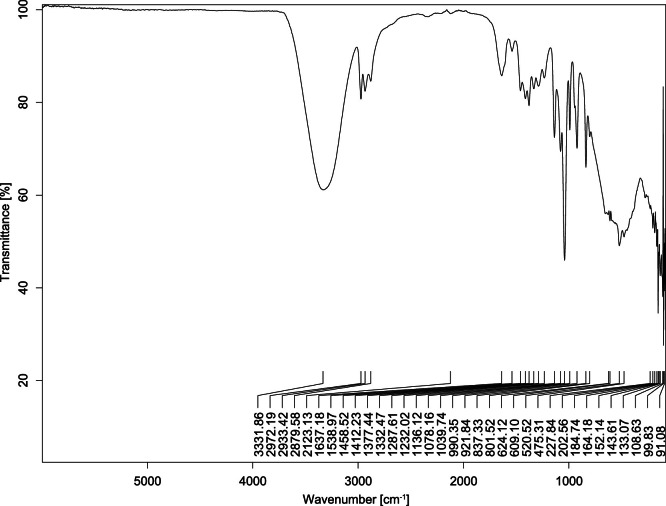
FTIR for LV_542_OKT_5B.

**Fig. 7:**
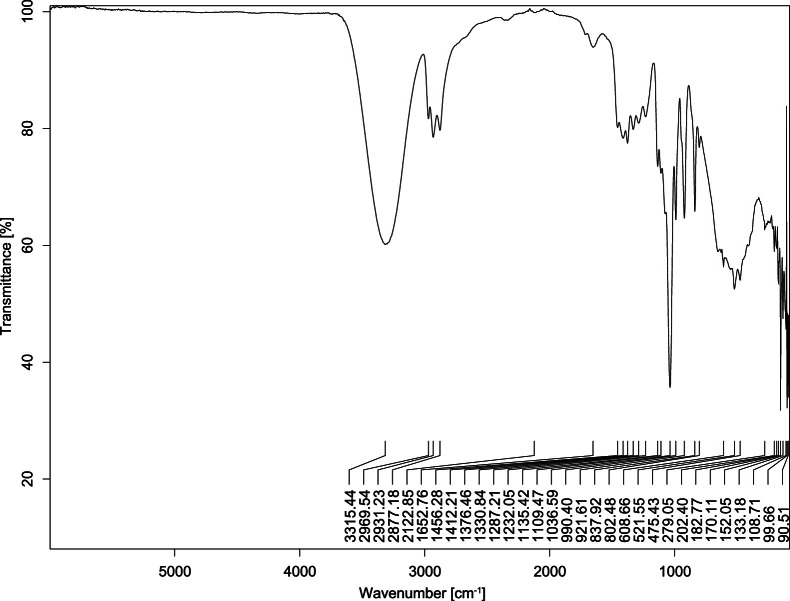
FTIR for LV_542_OKT_6.

**Fig. 8:**
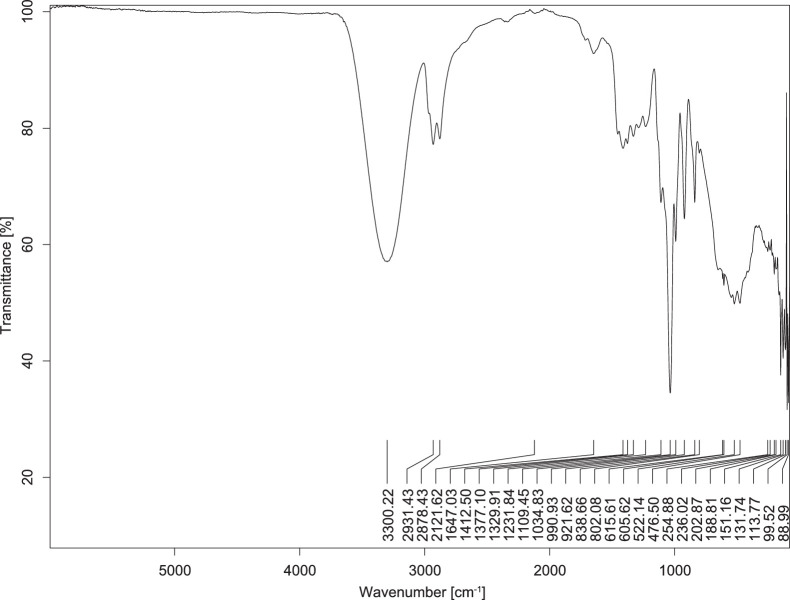
FTIR for LV_542_OKT_7.

**Fig. 9:**
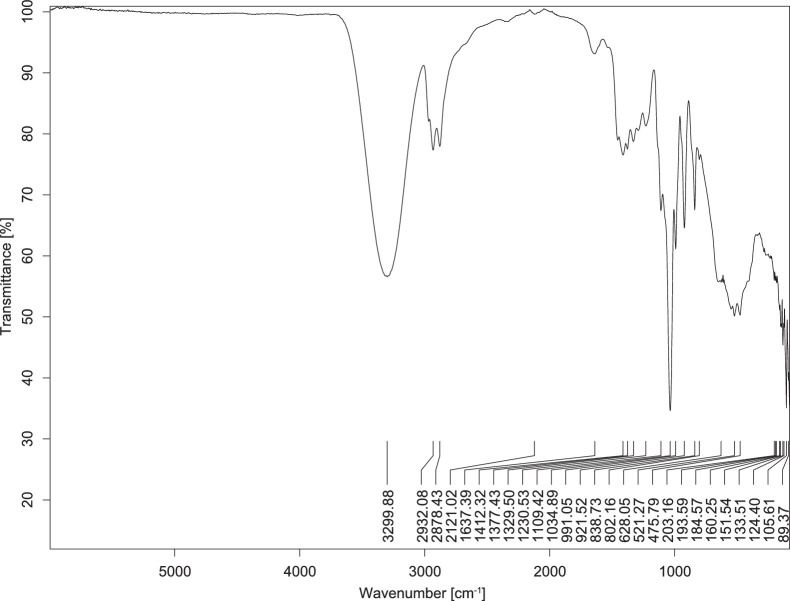
FTIR for LV_542_OKT_8.

**Fig. 10:**
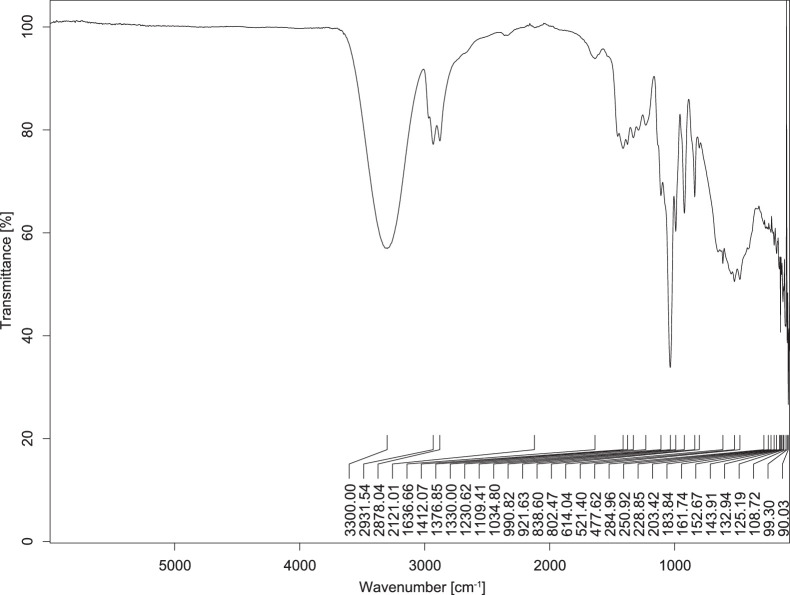
FTIR for LV_542_OKT_9.

**Fig. 11:**
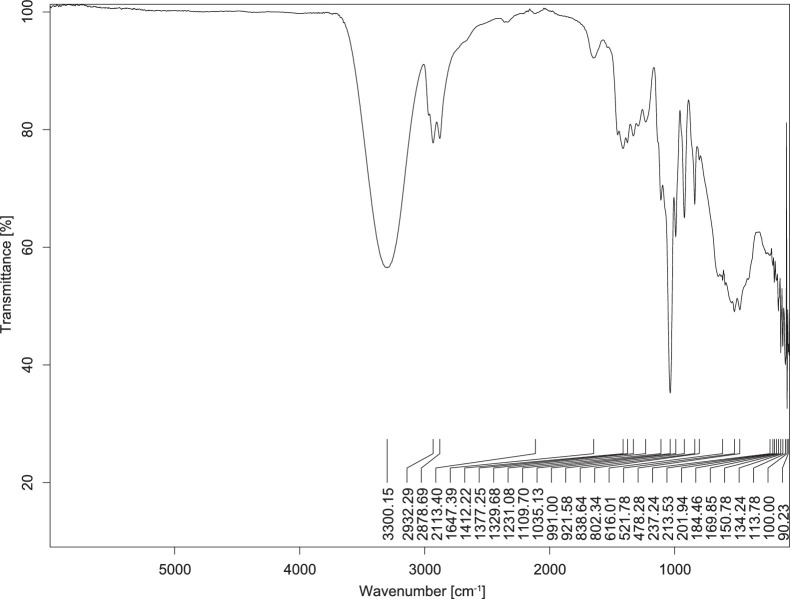
FTIR for LV_542_OKT_10.

**Fig. 12:**
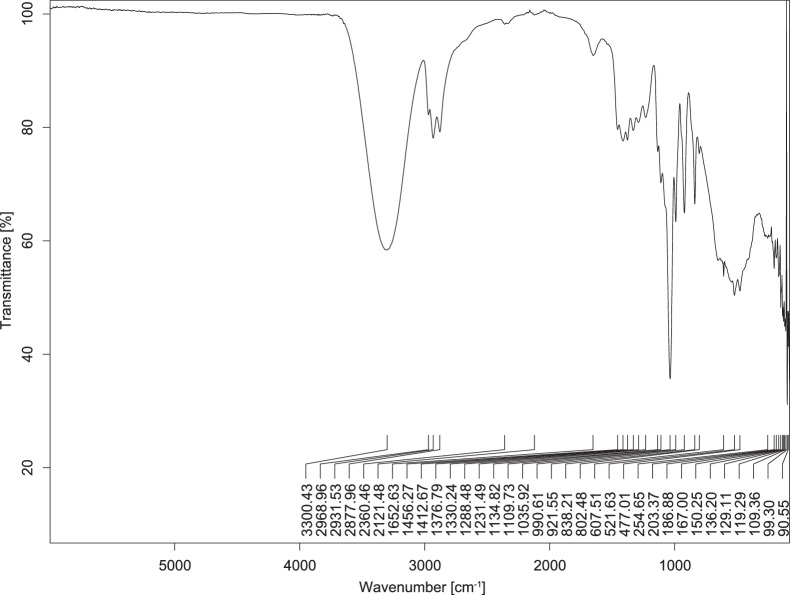
FTIR for LV_542_OKT_11.

**Fig. 13:**
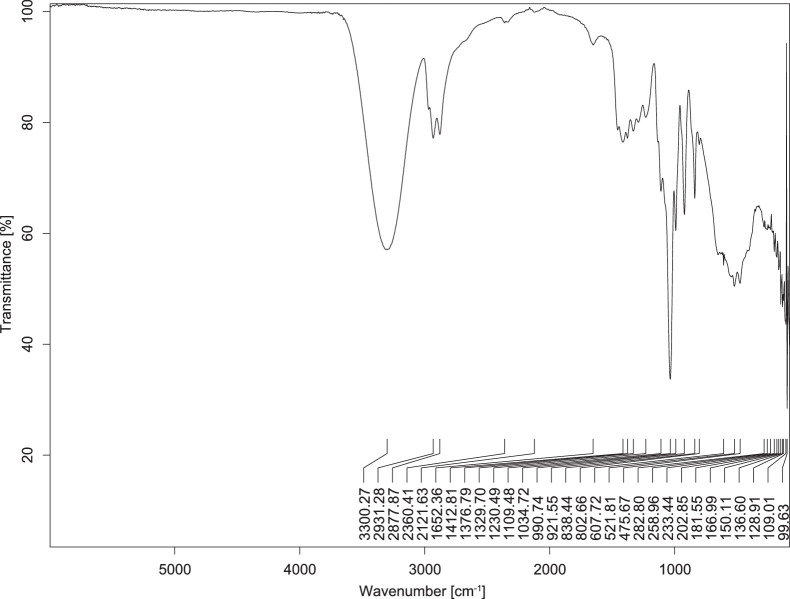
FTIR for LV_542_OKT_12.

**Fig. 14:**
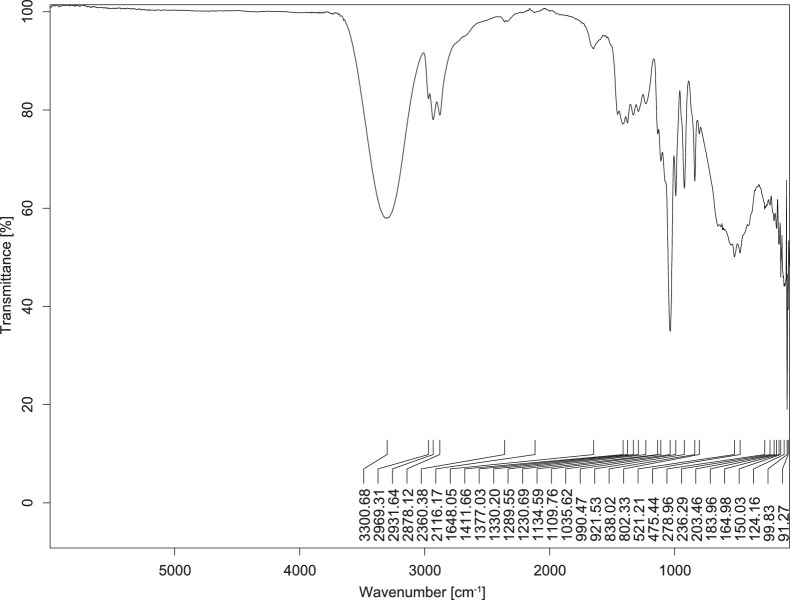
FTIR for LV_542_OKT_13.

**Fig. 15:**
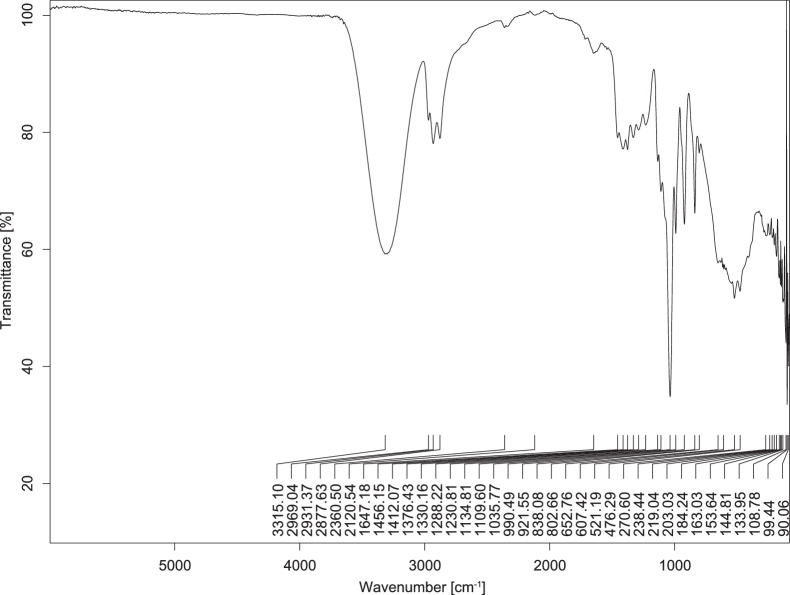
FTIR for LV_542_OKT_14.

**Fig. 16:**
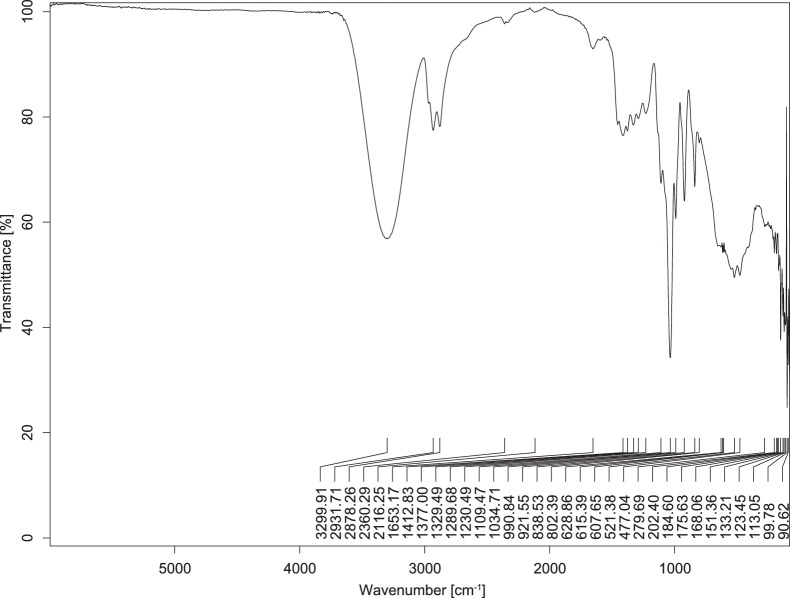
FTIR for LV_542_OKT_15.

**Fig. 17:**
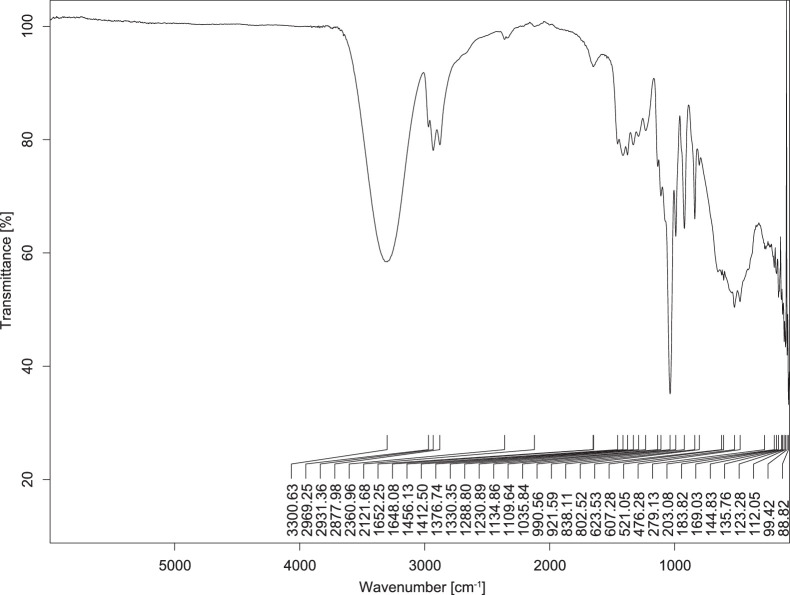
FTIR for LV_542_OKT_16.

**Fig. 18:**
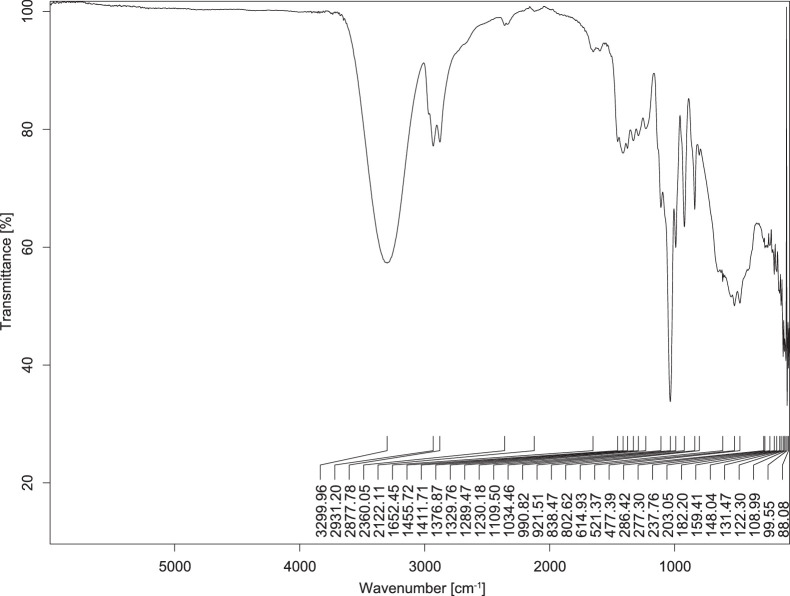
FTIR for LV_542_OKT_17.

**Fig. 19:**
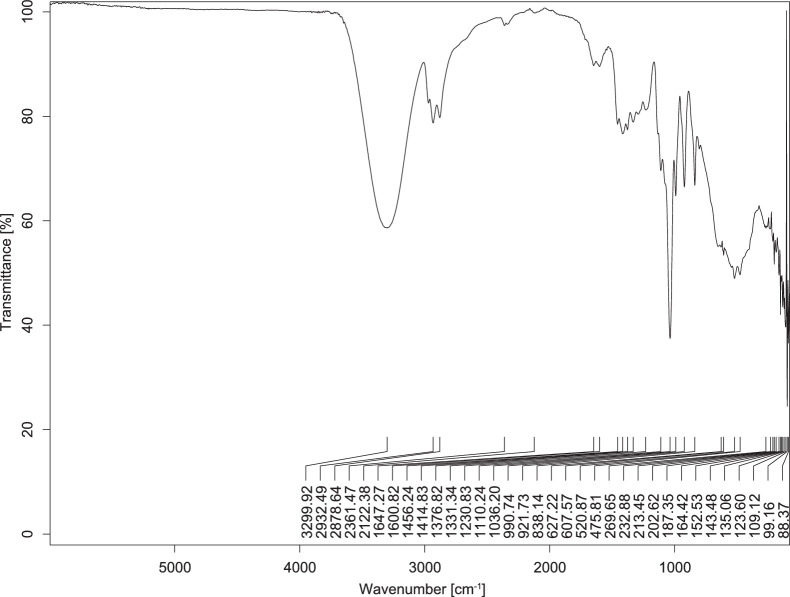
FTIR for LV_542_OKT_18.

**Fig. 20:**
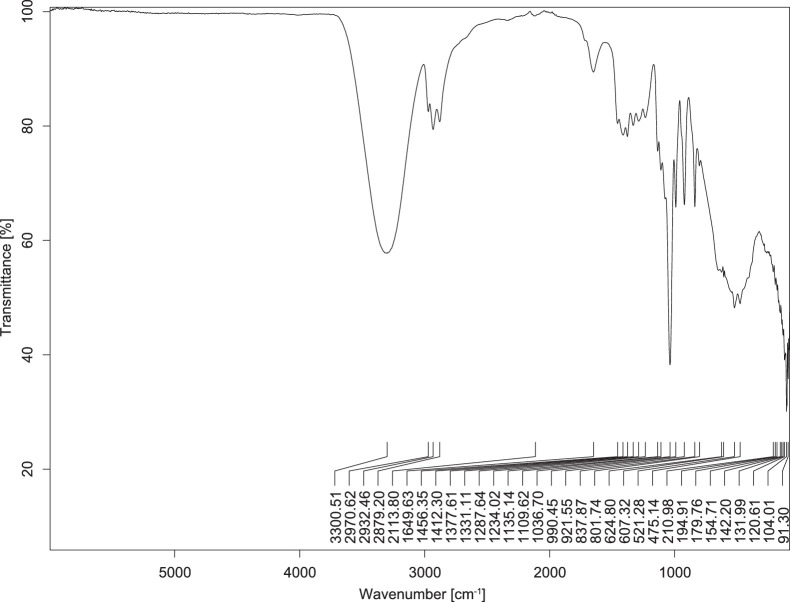
FTIR for LV_542_OKT_19.

**Fig. 21:**
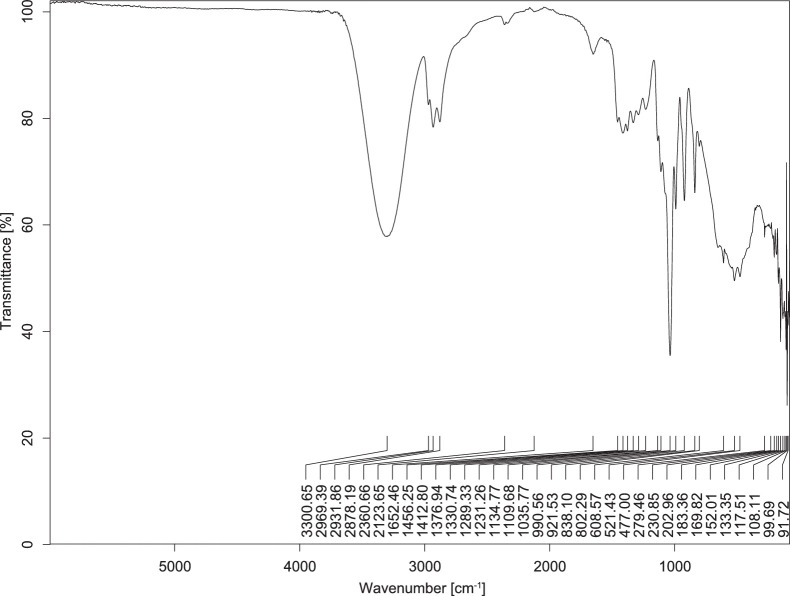
FTIR for LV_542_OKT_20.

**Fig. 22:**
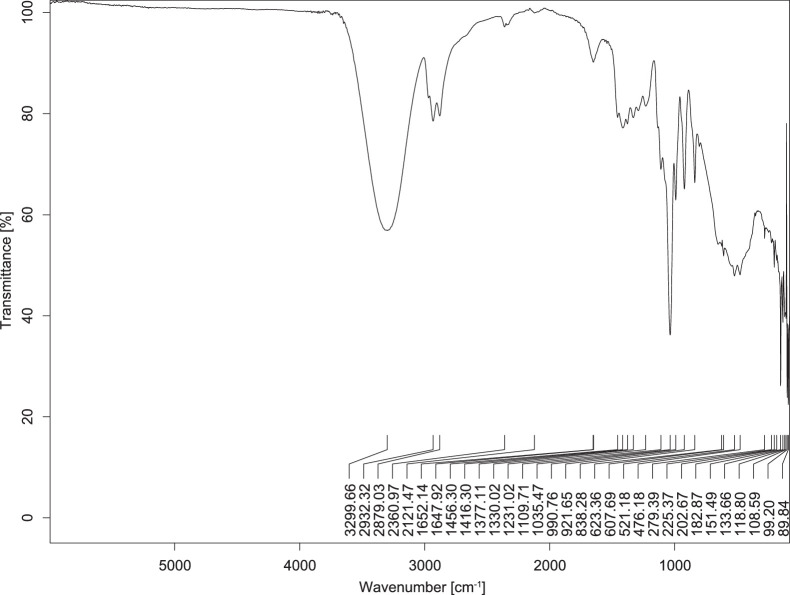
FTIR for LV_542_OKT_21.

**Fig. 23:**
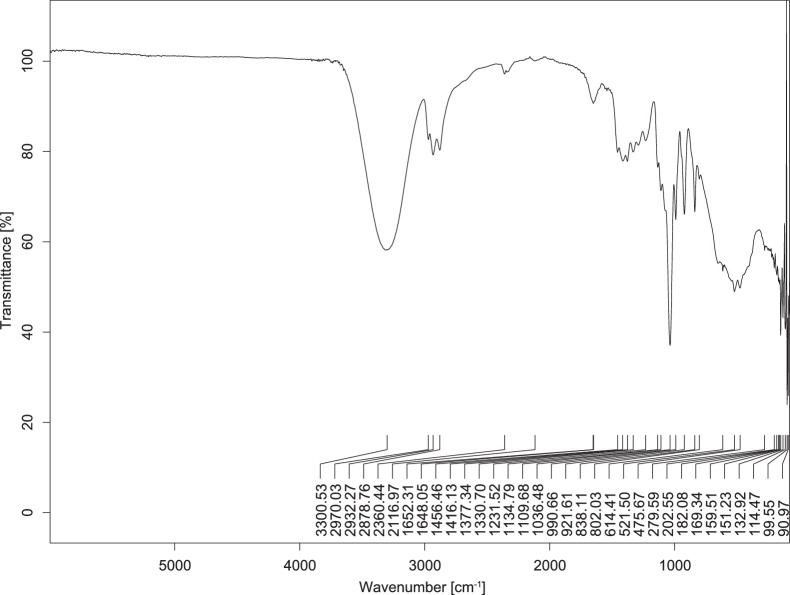
FTIR for LV_542_OKT_22.

**Fig. 24:**
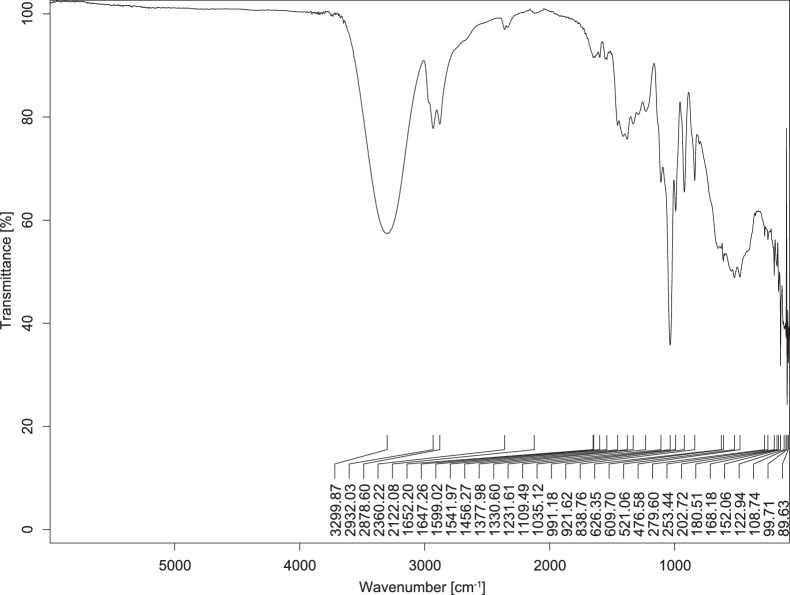
FTIR for LV_542_OKT_23.

**Fig. 25:**
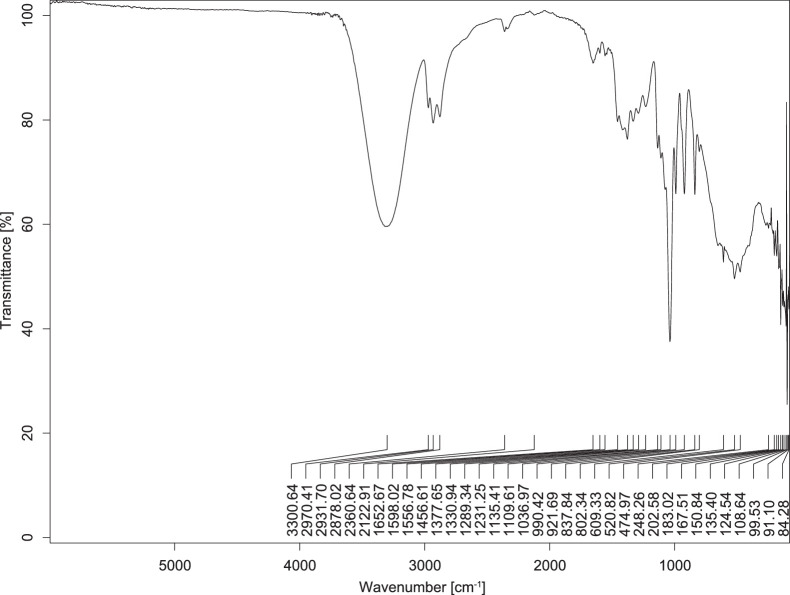
FTIR for LV_542_OKT_24.

**Fig. 26:**
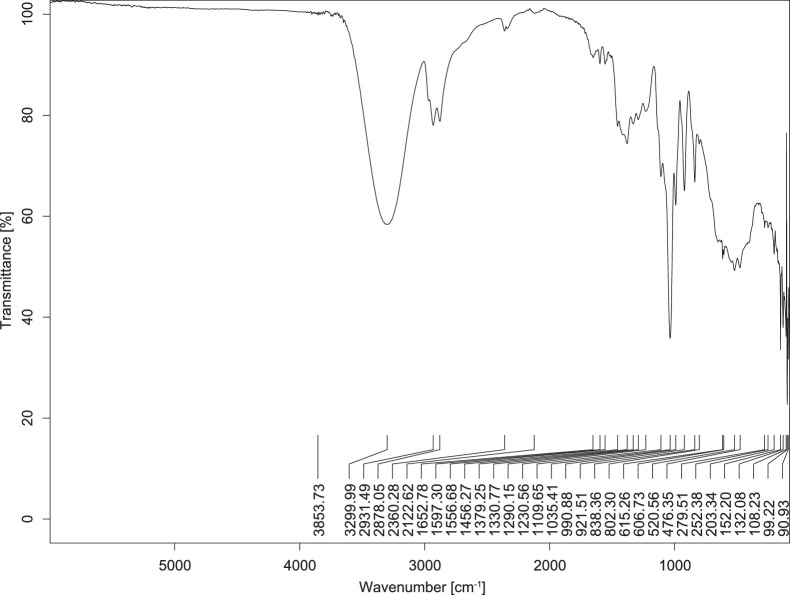
FTIR for LV_542_OKT_25.

**Fig. 27:**
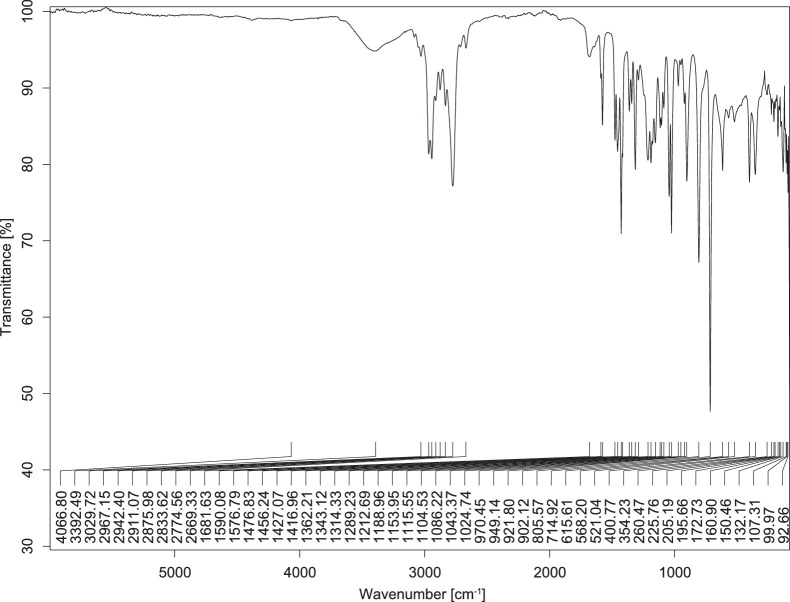
FTIR for NIC_IPIN_MAY.

None of the samples exhibited a signal at 714 cm^−1^. This region is hindered by a broad absorption band containing several distinct peaks centred around 600 cm^−1^. Due to the complexity of the matrix, significant peak overlapping is expected. The vibrational modes typically associated with this region include C-H bending of aromatic and alkene groups, as well as C-Cl stretching. This region is not unique to nicotine but is also relevant to other e-liquid components.

Table 3 presents the potential peak interferences of nicotine by propylene glycol (PG) and vegetable glycerine (VG). At approximately 3300 cm^−1^, the N-H stretching band of nicotine is predominantly overlapped by the strong and broad O-H stretching bands of PG and VG [2]. Similarly, the C-H stretching bands observed between 2800 and 3000 cm^−1^ are also expected to be overlapped by PG and VG [2]. The C-O stretching vibrations occurring between ∼1000 cm^−1^ and 1300 cm^−1^ may interfere with the C–N stretching band of nicotine at ∼1316 cm^−1^ [3]. In addition, the aromatic C=C stretching vibration of nicotine, typically observed at ∼1653 cm^−1^ [4], could potentially be obscured by signals related to organic acids, flavourings, and colourings, particularly C=O stretching and aromatic C=C stretching [[5], [6], [7], [8], [9], [10], [11]]. The peak corresponding to the C=N stretching of nicotine’s pyridine ring, expected at 1585 cm^−1^ [4], was not detected in any of the samples in the current study.

**Table 3:** Potential peak interferences of nicotine signals by PG and VG.

Component	Wavenumber (cm^−1^)												References
Nicotine	∼3500-3300 (N-H stretch)	-	∼3000-2800 (C-H stretch)	-	-	∼1653 (C=C stretch, aromatic)	∼1585 (C=N stretch, aromatic)	-	∼1316 (C-N stretch, aromatic)	-	∼904 (C-H bend, out-of-plane)	∼712 (C-H bend, out-of-plane)	[3,4,12]
PG	3302 (O-H stretch)	-	2877 (C-H stretch)	-	-	-	-	-	-	1036 (C-O stretch)	-	-	[2]
VG	3268 (O-H stretch)	-	2877 (C-H stretch)	-	-	-	-	-	-	1028 (C-O stretch)	-	-	[2]

"-" indicates the absence of signal(s) representing the said compound, as reported by previous studies.

However, a peak at approximately 805 cm^−1^ – close to the 714 cm^−1^ region – was consistently observed across all 25 e-liquid samples and the e-liquid booster. This signal, which is also present in the NIC_IPIN_MAY spectrum, may be attributed to nicotine [12].

The NMR Data repository includes raw free induction decay (FID) files and processed spectra (1r) in Bruker format, these data can be opened with any NMR software (e.g. Topspin, Mnova etc.). The ^1^H NMR spectra provided a global fingerprint of the sample composition including the matrix, nicotine and additives.

**Fig. 28:**
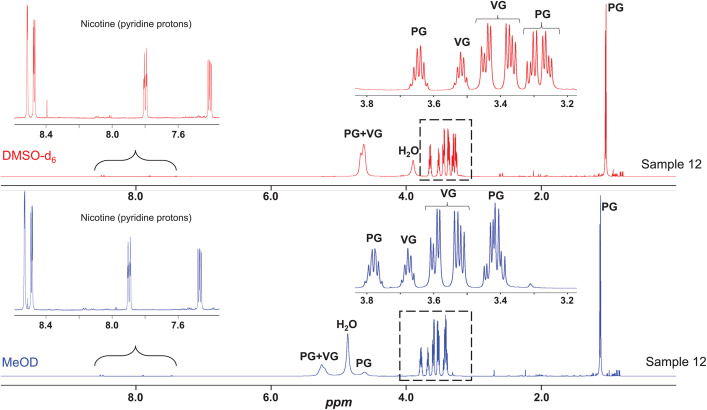
^1^H NMR spectra of sample 12 (LV_542_OKT_11). DMSO-d_6_ = dimethyl sulfoxide-d_6_ and MeOD = methanol-d_4_.

In Fig. 28 below are the typical profiles obtained in both solvents (dimethyl sulfoxide-d_6_ and methanol-d_4_) for sample 12 (LV_542_OKT_11). The dominant signals were from the matrix components, i.e. PG and VG. In methanol-d_4_ (MeOD), for PG, a doublet (CH_3_) was detected at 1.12 ppm (J = 6.7 Hz), a multiplet (CH) at 3.77 ppm, an ABd system (CH_2_) centered at 3.41 ppm (J = 11.1, 6.5, 4.6 Hz), and broad signals for hydroxyl protons at 4.61 and 5.24 ppm. The methine and methylene groups of VG give rise to a multiplet at 3.67 ppm and an ABd system at 3.59 and 3.52 ppm (J = 11.3, 6.1, 4.8 Hz), respectively, while the hydroxyl groups resonate as a broad signal at 5.24 ppm, overlapping with one hydroxyl signal of PG. The nicotine signals are minor signals compared to those of the matrix. However, a zoomed-in region (7.4–8.2 ppm) highlights the presence of pyridine proton signals. Apart from small variations in chemical shifts, no significant differences are observed between the two solvents. Both solvents can be effectively used for sample profiling.

**Fig. 29:**
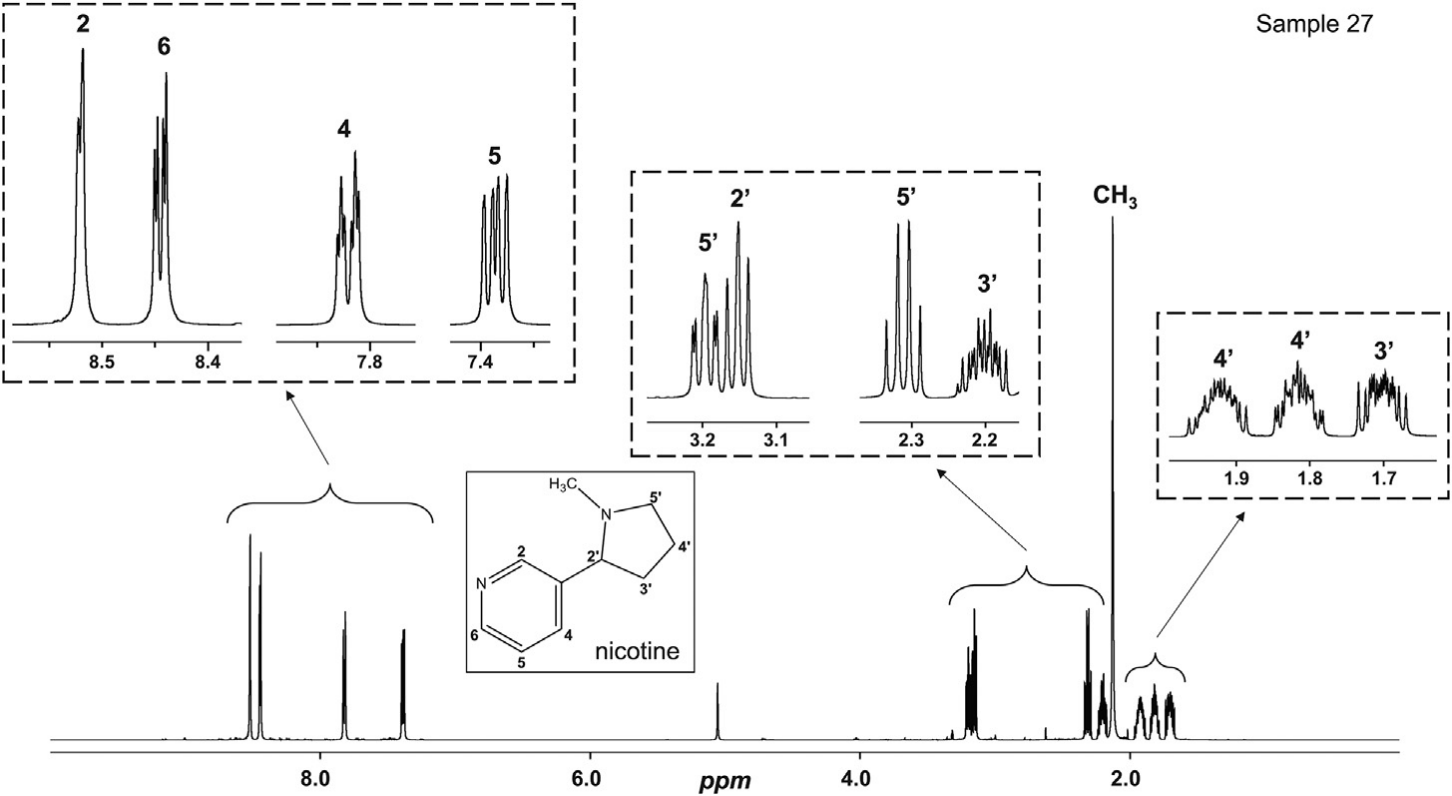
^1^H NMR spectrum of sample 27 (NIC_IPIN_MAY) in MeOD.

Sample 27 (NIC_IPIN_MAY), a nicotine solution, allows clear visualisation of the signals without matrix interference. Signal assignments are reported on the spectrum (Fig. 29).

**Fig. 30:**
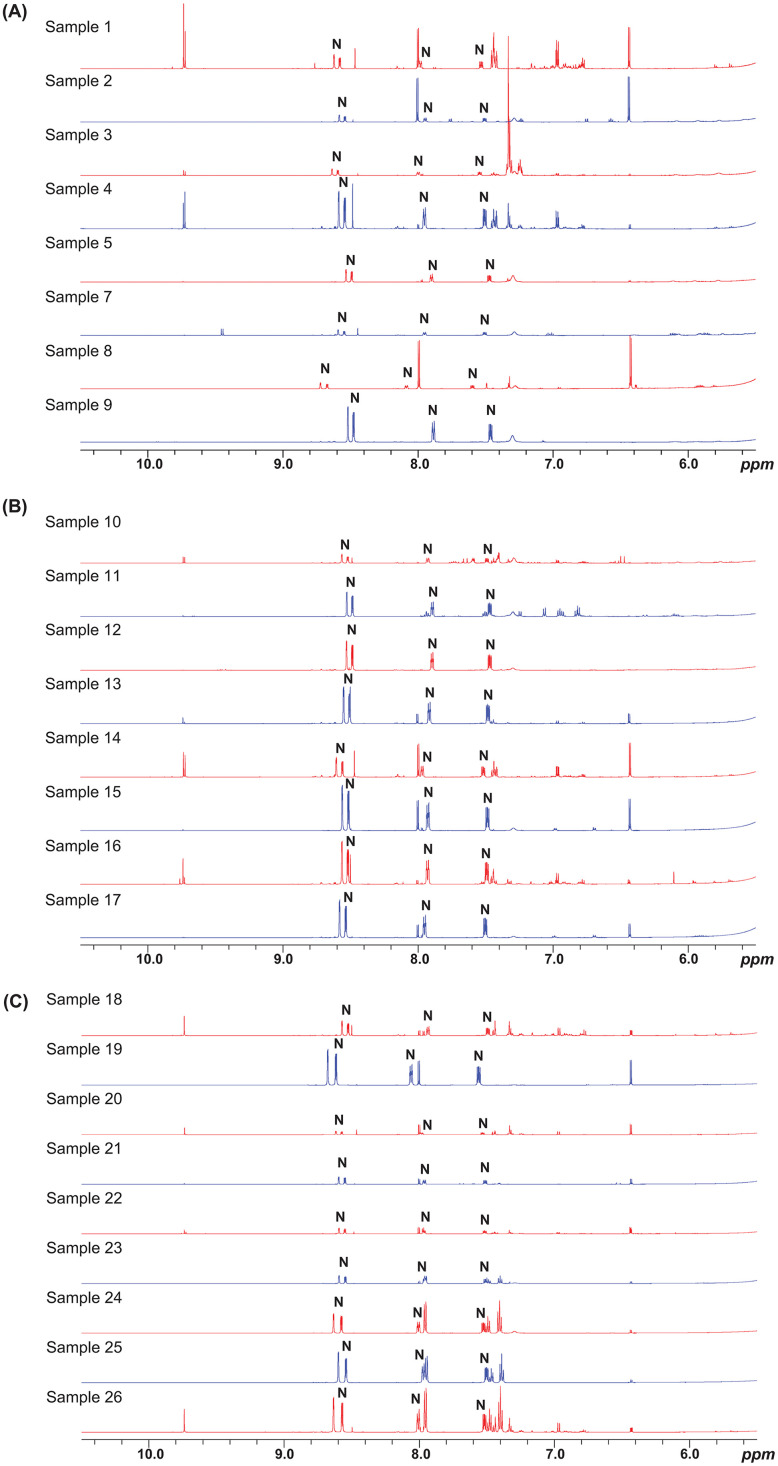
^1^H NMR profiling in MeOD of the 25 e-liquid samples (Zoom 5.5-10.5 ppm).

Fig. 30 below compares the ^1^H NMR of the 25 e-liquid samples, focusing on the spectral window between 5.5 and 10.5 ppm. This zoomed-in region highlights spectral differences between the samples, allowing for the detection of nicotine signals (N) as well as signals from flavouring compounds or other additives. Variations in the signal intensity of nicotine reflect differences in nicotine concentration, while slight chemical shift variations can be attributed to the differences in nicotine form (salt or freebase). These ¹H NMR spectral differences, observed in both nicotine and other compounds, highlight variations in sample compositions and emphasise the ability of NMR to differentiate them [13].

The generated chromatograms of e-liquid samples are shown between Fig. 31, Fig. 32, Fig. 33, Fig. 34, Fig. 35, Fig. 36, Fig. 37, Fig. 38, Fig. 39, Fig. 40, Fig. 41, Fig. 42, Fig. 43, Fig. 44, Fig. 45, Fig. 46, Fig. 47, Fig. 48, Fig. 49, Fig. 50, Fig. 51, Fig. 52, Fig. 53, Fig. 54, Fig. 55, Fig. 56-56. Meanwhile, the nicotine solution chromatogram is shown in Fig. 57. The x-axis represents the retention time in minutes, indicating the time each compound takes to pass through the chromatographic column, while the y-axis displays the abundance, corresponding to the intensity of the detected ion signals.

**Fig. 31:**
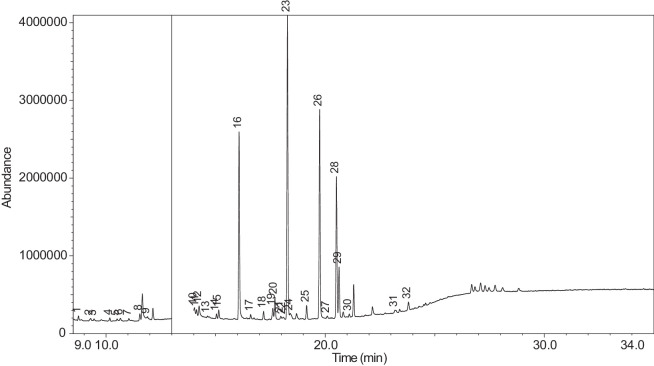
Chromatogram for LV_542_OKT_1. The peak numbers are referred to as follows:1 = Propylene Glycol; 2 = Methyl propyl ether; 3 = 2-Propanol, 1,3-dichloro-; 4 = 1,2-Propanediol, 1-acetate; 5 = 1,2-Propanediol, 2-acetate; 6 = Cyclotetrasiloxane, octamethyl-; 7 = Oxime-, methoxy-phenyl-; 8 = 1,3-Dioxolane, 2,2,4-trimethyl-, 9 = Methyl propionate; 10 = Phenol, 2-methoxy-; 11 = 2(3H)-Furanone, 5-ethyldihydro-; 12 = Glycerin; 13 = Maltol; 14 = Benzene, 1,4-dimethoxy-; 15 = 1,2,3-Propanetriol, 1-acetate; 16 = Ethyl maltol; 17 = Alpha-monopropionin; 18 = Benzaldehyde, 4-methoxy-; 19 = Benzenemethanol, 4-methoxy-; 20 = 5-Thiazoleethanol, 4-methyl-; 21 = 2,3-dihydroxypropyl isobutyrate; 22 = 1,4-Dioxane-2,6-dimethanol; 23 = Pyridine, 3-(1-methyl-2-pyrrolidinyl)-, (S)-; 24 = p-Dioxane-2,5-dimethanol; 25 = 2(3H)-Furanone, dihydro-5-pentyl-; 26 = Benzaldehyde, 3-hydroxy-4-methoxy-; 27 = 1,4-Benzenediol, 2-methoxy-; 28 = Ethyl Vanillin; 29 = Mandelic acid, 3,4-dimethoxy-, methyl ester; 30 = 2H-Pyran-2-one, tetrahydro-6-pentyl-; 31 = Vanillin propylene glycol acetal; 32 = .delta.-Dodecalactone.

**Fig. 32:**
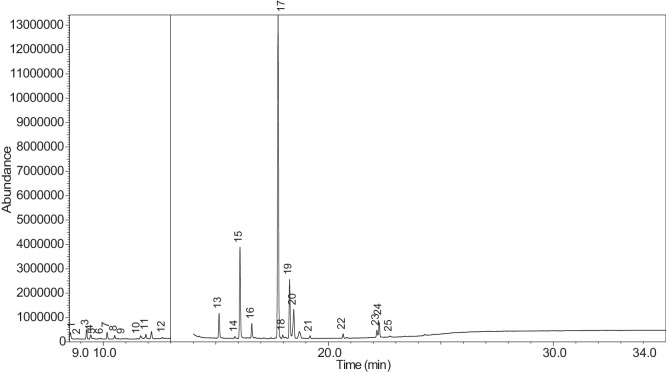
Chromatogram for LV_542_OKT_2. The peak numbers are referred to as follows:1 = Butanoic acid, 2-methyl-, ethyl ester; 2 = Ethanedioic acid, dimethyl ester; 3 = 3-Hexen-1-ol, (Z)-; 4 = 1-Hexanol; 5 = Butanoic acid, 2-methyl-; 6 = Acetic acid, pentyl ester; 7 = 1,2-Propanediol, 1-acetate; 8 = 1,2-Propanediol, 2-acetate; 9 = 1,3-Dioxolane-2-methanol, 2,4-dimethyl-; 10 = Hexanoic acid, ethyl ester; 11 = Methyl propionate; 12 = Butanoic acid, 3-methylbutyl ester; 13 = 1,2,3-Propanetriol, 1-acetate; 14 = 1,2-Ethanediol, diacetate; 15 = Ethyl maltol; 16 = Alpha-monopropionin; 17 = .alpha.-d-Erythro-hex-2-enopyranoside, ethyl 2,3-dideoxy-; 18 = 2,3-dihydroxypropyl isobutyrate; 19 = Pyridine, 3-(1-methyl-2-pyrrolidinyl)-, (S)-; 20 = Methyl anthranilate; 21 = 2-Buten-1-one, 1-(2,6,6-trimethyl-1-cyclohexen-1-yl)-; 22 = 2(3H)-Furanone, 5-hexyldihydro-; 23 = p-Dioxane-2,5-dimethanol; 24 = Cyclohexanecarboxamide, N-ethyl-5-methyl-2-(1-methylethyl)-; 25 = 1,4-Dioxane-2,6-dimethanol.

**Fig. 33:**
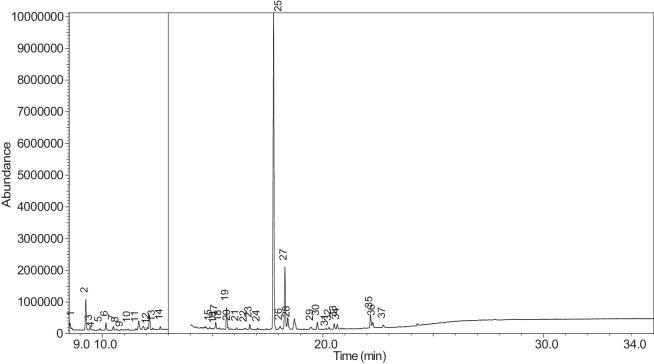
Chromatogram for LV_542_OKT_3. The peak numbers are referred to as follows: 1 = Butanoic acid, 2-methyl-, ethyl ester; 2 = 3-Hexen-1-ol, (Z)-; 3 = 2-Propanol, 1,3-dichloro-; 4 = Butanoic acid, 2-methyl-; 5 = Acetic acid, pentyl ester; 6 = 1,2-Propanediol, 1-acetate; 7 = 1,2-Propanediol, 2-acetate; 8 = Cyclotetrasiloxane, octamethyl-; 9 = 1,3-Dioxolane-2-methanol, 2,4-dimethyl-; 10 = (R)-(-)-2,2-Dimethyl-1,3-dioxolane-4-methanol; 11 = Hexanoic acid, ethyl ester; 12 = D-Limonene; 13 = Eucalyptol; 14 = Butanoic acid, 3-methylbutyl ester; 15 = Cyclohexanol, 1-methyl-4-(1-methylethenyl)-; 16 = Octanoic acid, ethyl ester; 17 = 1,2,3-Propanetriol, 1-acetate; 18 = 3-Cyclohexen-1-ol, 4-methyl-1-(1-methylethyl)-, (R)-; 19 = L-.alpha.-Terpineol; 20 = Cyclohexanol, 1-methyl-4-(1-methylethylidene)-; 21 = 2,6-Octadien-1-ol, 3,7-dimethyl-, (Z)-; 22 = 1,3-Dioxolane, 2-heptyl-4-methyl-; 23 = (-)-Carvone; 24 = 1,2,3-Propanetriol, 1-acetate; 25 = .alpha.-d-Erythro-hex-2-enopyranoside, ethyl 2,3-dideoxy-; 26 = Triacetin; 27 = Pyridine, 3-(1-methyl-2-pyrrolidinyl)-, (S)-; 28 = Pyridine, 3-(1-methyl-2-pyrrolidinyl)-, (S)-; 29 = .alpha.-Ionone; 30 = Benzaldehyde, 3-hydroxy-4-methoxy-; 31 = Butylated Hydroxytoluene; 32 = trans-.beta.-Ionone; 33 = Ethyl Vanillin; 34 = 2(3H)-Furanone, 5-hexyldihydro-; 35 = p-Dioxane-2,5-dimethanol; 36 = Cyclohexanecarboxamide, N-ethyl-5-methyl-2-(1-methylethyl)-; 37 = p-Dioxane-2,5-dimethanol.

**Fig. 34:**
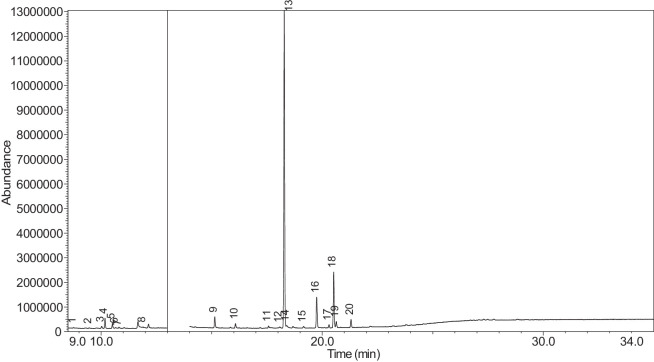
Chromatogram for LV_542_OKT_4. The peak numbers are referred to as follows: 1 = Propylene Glycol; 2 = 2-Propanol, 1,3-dichloro-; 3 = 2-Furanmethanol, tetrahydro-; 4 = 1,2-Propanediol, 1-acetate; 5 = 1,2-Propanediol, 2-acetate; 6 = Cyclotetrasiloxane, octamethyl-; 7 = 1,2-Propanediol, 3-methoxy-; 8 = Methyl propionate; 9 = 1,2,3-Propanetriol, 1-acetate; 10 = Ethyl maltol; 11 = 2(3H)-Furanone, 5-butyldihydro-; 12 = 1,4-Dioxane-2,6-dimethanol; 13 = Pyridine, 3-(1-methyl-2-pyrrolidinyl)-, (S)-; 14 = p-Dioxane-2,5-dimethanol; 15 = 2(3H)-Furanone, dihydro-5-pentyl-; 16 = Benzaldehyde, 3-hydroxy-4-methoxy-; 17 = (1′s,2′s)-Nicotine-N'-oxide; 18 = Ethyl Vanillin; 19 = Mandelic acid, 3,4-dimethoxy-, methyl ester; 20 = Danielone.

**Fig. 35:**
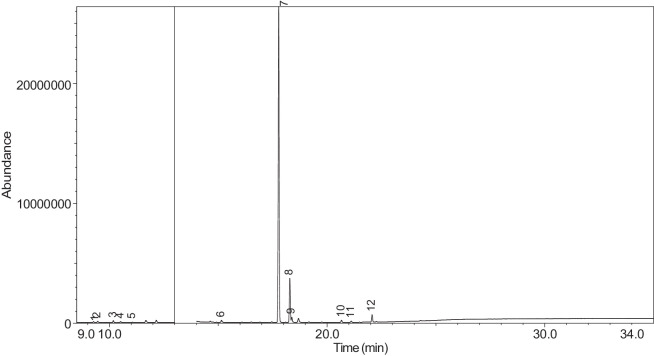
Chromatogram for LV_542_OKT_5A. The peak numbers are referred to as follows: 1 = 3-Hexen-1-ol, (Z)-; 2 = 2-Propanol, 1,3-dichloro-; 3 = 1,2-Propanediol, 1-acetate; 4 = 1,2-Propanediol, 2-acetate; 5 = Oxime-, methoxy-phenyl-; 6 = 1,2,3-Propanetriol, 1-acetate; 7 = .alpha.-d-Erythro-hex-2-enopyranoside, ethyl 2,3-dideoxy-; 8 = Pyridine, 3-(1-methyl-2-pyrrolidinyl)-, (S)-; 9 = Pyridine, 3-(1-methyl-2-pyrrolidinyl)-, (S)-; 10 = 2(3H)-Furanone, 5-hexyldihydro-; 11 = 2H-Pyran-2-one, tetrahydro-6-pentyl-; 12 = 2(3H)-Furanone, 5-heptyldihydro-.

**Fig. 36:**
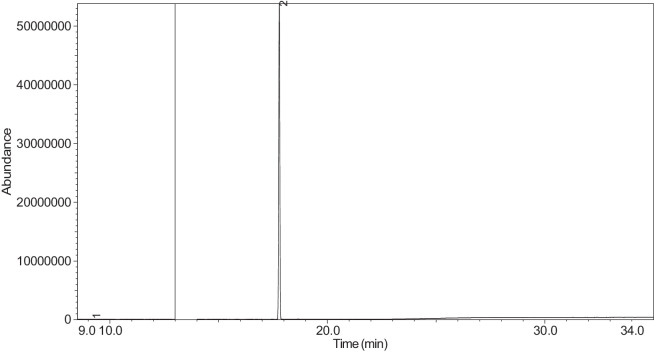
Chromatogram for LV_542_OKT_5B. The peak numbers are referred to as follows: 1 = 2-Propanol, 1,3-dichloro-; 2 = Hexanamide, 3,5,5-trimethyl-N-pentyl-.

**Fig. 37:**
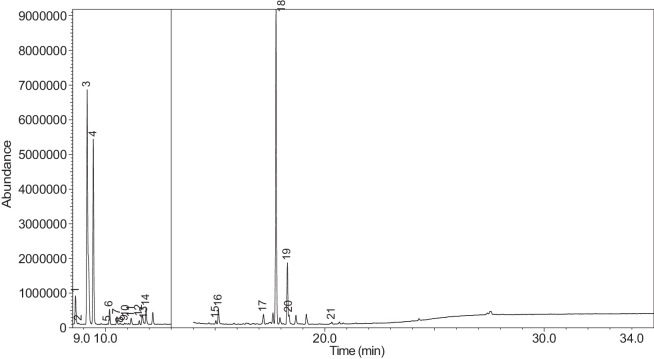
Chromatogram for LV_542_OKT_6. The peak numbers are referred to as follows: 1 = Butanoic acid, 3-methyl-, ethyl ester; 2 = Propylene Glycol; 3 = 1-Butanol, 3-methyl-, acetate; 4 = 1-Hexanol; 5 = 3-Methoxyhex-1-ene; 6 = 1,2-Propanediol, 1-acetate; 7 = 1,2-Propanediol, 2-acetate; 8 = Cyclotetrasiloxane, octamethyl-; 9 = 1,3-Dioxolane-2-methanol, 2,4-dimethyl-; 10 = 1,3-Dioxolane-2-methanol, 2,4-dimethyl-; 11 = trans-2-Hexenal dimethyl acetal; 12 = Hexanoic acid, ethyl ester; 13 = 3-Hexen-1-ol, acetate, (E)-; 14 = Acetic acid, hexyl ester; 15 = Butanoic acid, hexyl ester; 16 = 1,2,3-Propanetriol, 1-acetate; 17 = 3-Pentanol, 2,3,4-trimethyl-; 18 = .alpha.-d-Erythro-hex-2-enopyranoside, ethyl 2,3-dideoxy-; 19 = Pyridine, 3-(1-methyl-2-pyrrolidinyl)-, (S)-; 20 = Pyridine, 3-(1-methyl-2-pyrrolidinyl)-, (S)-; 21 = (1′s,2′s)-Nicotine-N'-oxide.

**Fig. 38:**
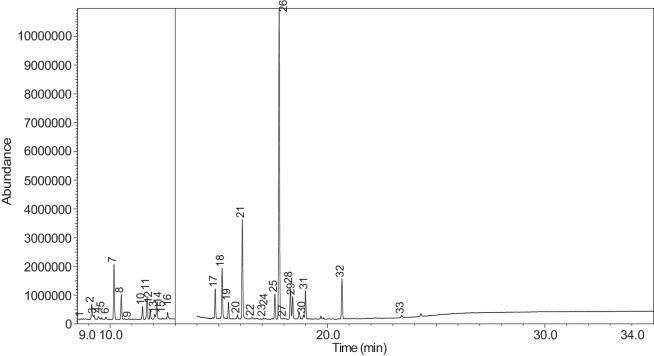
Chromatogram for LV_542_OKT_7. The peak numbers are referred to as follows: 1 = 1,3-Dioxolane-2-methanol, 2,4-dimethyl-; 2 = 1-Butanol, 3-methyl-, acetate; 3 = 3-Hexen-1-ol, (E)-; 4 = 2-Propanol, 1,3-dichloro-; 5 = 2-Furanmethanol; 6 = Dimethyl Sulfoxide; 7 = 1,2-Propanediol, 1-acetate; 8 = 1,2-Propanediol, 2-acetate; 9 = 1,3-Dioxolane-2-methanol, 2,4-dimethyl-; 10 = Butanoic acid, butyl ester; 11 = 2-Furanmethanol, acetate; 12 = Acetic acid, hexyl ester; 13 = D-Limonene; 14 = 1,3,6-Octatriene, 3,7-dimethyl-, (Z)-; 15 = 1,2-Propanediol, diacetate; 16 = Butanoic acid, 3-methylbutyl ester; 17 = Allyl heptanoate; 18 = 1,2,3-Propanetriol, 1-acetate; 19 = Benzenemethanol, .alpha.-methyl-, acetate; 20 = 1,2,3-Propanetriol, 1-acetate; 21 = Ethyl maltol; 22 = 2,3-Butanedione, monoxime; 23 = 1,2,3-Propanetriol, 1-acetate; 24 = Glycerol 1,2-diacetate; 25 = 2(3H)-Furanone, 5-butyldihydro-; 26 = .alpha.-d-Erythro-hex-2-enopyranoside, ethyl 2,3-dideoxy-; 27 = 2,3-dihydroxypropyl isobutyrate; 28 = Pyridine, 3-(1-methyl-2-pyrrolidinyl)-, (S)-; 29 = Pyridine, 3-(1-methyl-2-pyrrolidinyl)-, (S)-; 30 = Diphenyl ether; 31 = Cyclohexanepropanoic acid, 2-propenyl ester; 32 = 2(3H)-Furanone, 5-hexyldihydro-; 33 = Glycerin, 1,2-dicaprylate.

**Fig. 39:**
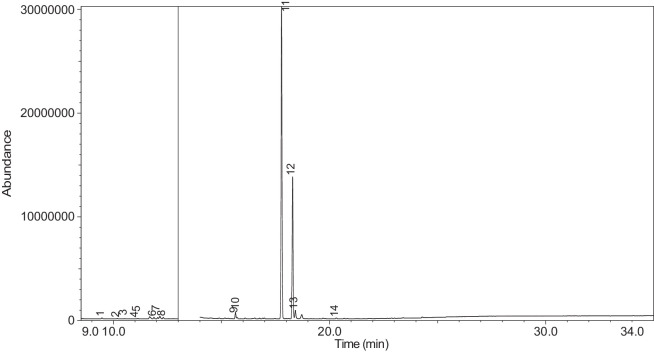
Chromatogram for LV_542_OKT_8. The peak numbers are referred to as follows: 1 = 2-Propanol, 1,3-dichloro-; 2 = 1,2-Propanediol, 1-acetate; 3 = 1,2-Propanediol, 2-acetate; 4 = Oxime-, methoxy-phenyl-; 5 = Bicyclo[3.1.1]heptane, 6,6-dimethyl-2-methylene-, (1S)-; 6 = 7-Oxabicyclo[2.2.1]heptane, 1-methyl-4-(1-methylethyl)-; 7 = D-Limonene; 8 = Eucalyptol; 9 = L-.alpha.-Terpineol; 10 = Cyclohexanol, 1-methyl-4-(1-methylethylidene)-; 11 = .alpha.-d-Erythro-hex-2-enopyranoside, ethyl 2,3-dideoxy-; 12 = Pyridine, 3-(1-methyl-2-pyrrolidinyl)-, (S)-; 13 = Pyridine, 3-(1-methyl-2-pyrrolidinyl)-, (S)-; 14 = (1′s,2′s)-Nicotine-N'-oxide.

**Fig. 40:**
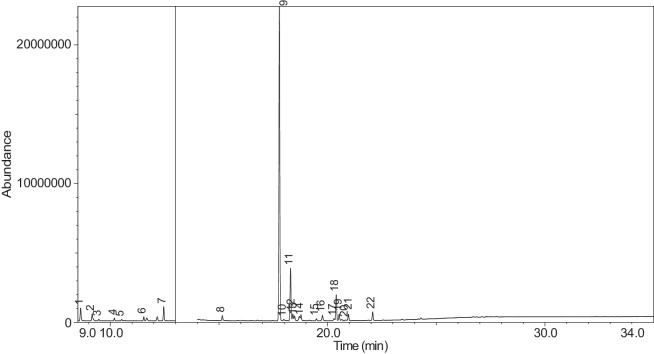
Chromatogram for LV_542_OKT_9. The peak numbers are referred to as follows: 1 = Butanoic acid, 3-methyl-, ethyl ester; 2 = 1-Butanol, 3-methyl-, acetate; 3 = 2-Propanol, 1,3-dichloro-; 4 = 1,2-Propanediol, 1-acetate; 5 = 1,2-Propanediol, 2-acetate; 6 = Hexanoic acid, ethyl ester; 7 = Butanoic acid, 3-methyl-, butyl ester; 8 = 1,2,3-Propanetriol, 1-acetate; 9 = .alpha.-d-Erythro-hex-2-enopyranoside, ethyl 2,3-dideoxy-; 10 = 2,3-dihydroxypropyl isobutyrate; 11 = Pyridine, 3-(1-methyl-2-pyrrolidinyl)-, (S)-; 12 = Pyridine, 3-(1-methyl-2-pyrrolidinyl)-, (S)-; 13 = Methyl anthranilate; 14 = 2-Propenoic acid, 3-phenyl-, methyl ester; 15 = .alpha.-Ionone; 16 = Benzaldehyde, 3-hydroxy-4-methoxy-; 17 = 3-Buten-2-one, 4-(2,6,6-trimethyl-1-cyclohexen-1-yl)-; 18 = 2-Propenoic acid, 3-phenyl-, 1-methylethyl ester; 19 = Ethyl Vanillin; 20 = Pentanoic acid, 4-oxo-, butyl ester; 21 = Naphthalene, 2-ethoxy-; 22 = 2(3H)-Furanone, 5-heptyldihydro-.

**Fig. 41:**
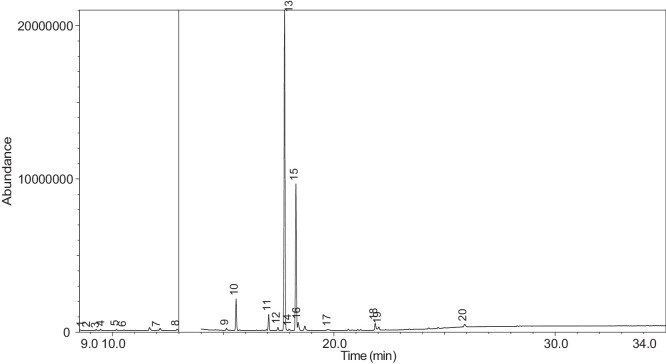
Chromatogram for LV_542_OKT_10. The peak numbers are referred to as follows: 1 = Butanoic acid, 2-methyl-, ethyl ester; 2 = Ethanedioic acid, dimethyl ester; 3 = 3-Hexen-1-ol, (Z)-; 4 = 2-Propanol, 1,3-dichloro-; 5 = 1,2-Propanediol, 1-acetate; 6 = 1,2-Propanediol, 2-acetate; 7 = D-Limonene; 8 = Phenol; 9 = 1,2,3-Propanetriol, 1-acetate; 10 = Benzene, 1-methoxy-4-propyl-; 11 = Anethole; 12 = (Z)-3-Phenylacrylaldehyde; 13 = .alpha.-d-Erythro-hex-2-enopyranoside, ethyl 2,3-dideoxy-; 14 = 2,3-dihydroxypropyl isobutyrate; 15 = Pyridine, 3-(1-methyl-2-pyrrolidinyl)-, (S)-; 16 = Pyridine, 3-(1-methyl-2-pyrrolidinyl)-, (S)-; 17 = Benzaldehyde, 3-hydroxy-4-methoxy-; 18 = Benzoic acid, 2-hydroxy-, 2-propenyl ester; 19 = Benzoic acid, 2-hydroxy-, 1-methylethyl ester; 20 = Benzoic acid, 2-hydroxy-, 2-methylpropyl ester.

**Fig. 42:**
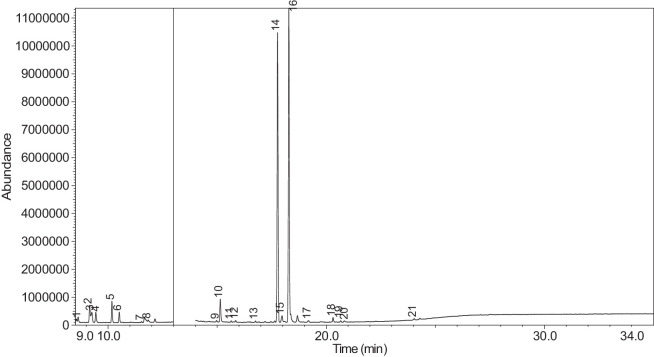
Chromatogram for LV_542_OKT_11. The peak numbers are referred to as follows: 1 = Butanoic acid, 2-methyl-, ethyl ester; 2 = 1-Butanol, 3-methyl-, acetate; 3 = 3-Hexen-1-ol; 4 = 1-Hexanol; 5 = 1,2-Propanediol, 1-acetate; 6 = 1,2-Propanediol, 2-acetate; 7 = Hexanoic acid, ethyl ester; 8 = Acetic acid, hexyl ester; 9 = Butanoic acid, 3-hexenyl ester, (Z)-; 10 = 1,2,3-Propanetriol, 1-acetate; 11 = L-.alpha.-Terpineol; 12 = 1,2,3-Propanetriol, 1-acetate; 13 = Propyl 2-ethylbutanoate; 14 = .alpha.-d-Erythro-hex-2-enopyranoside, ethyl 2,3-dideoxy-; 15 = 2,3-dihydroxypropyl isobutyrate; 16 = Pyridine, 3-(1-methyl-2-pyrrolidinyl)-, (S)-; 17 = 2-Buten-1-one, 1-(2,6,6-trimethyl-1-cyclohexen-1-yl)-; 18 = (1′s,2′s)-Nicotine-N'-oxide; 19 = 2(3H)-Furanone, dihydro-5-pentyl-; 20 = Propyl 2-methylvalerate; 21 = Benzyl Benzoate.

**Fig. 43:**
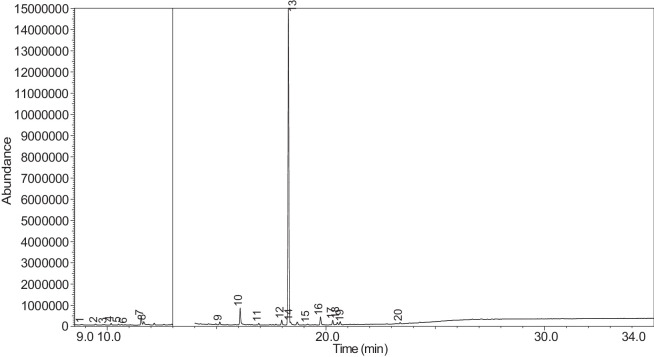
Chromatogram for LV_542_OKT_12. The peak numbers are referred to as follows: 1 = 3-Hexanol, 2-methyl-; 2 = 2-Propanol, 1,3-dichloro-; 3 = 2-Propenoic acid, 3-phenyl-, 2-methyl-2-propenyl ester; 4 = 1,2-Propanediol, 1-acetate; 5 = 1,2-Propanediol, 2-acetate; 6 = 1,2-Propanediol, 3-methoxy-; 7 = 1,3-Dioxolane, 2,2,4-trimethyl-; 8 = Silane, triethylmethoxy-; 9 = 1,2,3-Propanetriol, 1-acetate; 10 = Ethyl maltol; 11 = Cyclohexanol, 5-methyl-2-(1-methylethyl)-, acetate; 12 = 2,3-dihydroxypropyl isobutyrate; 13 = Pyridine, 3-(1-methyl-2-pyrrolidinyl)-, (S)-; 14 = Pyridine, 3-(1-methyl-2-pyrrolidinyl)-, (S)-; 15 = 2(3H)-Furanone, dihydro-5-pentyl-; 16 = Benzaldehyde, 3-hydroxy-4-methoxy-; 17 = (1′s,2′s)-Nicotine-N'-oxide; 18 = Ethyl Vanillin; 19 = 2(3H)-Furanone, 5-heptyldihydro-; 20 = .gamma.-Dodecalactone.

**Fig. 44:**
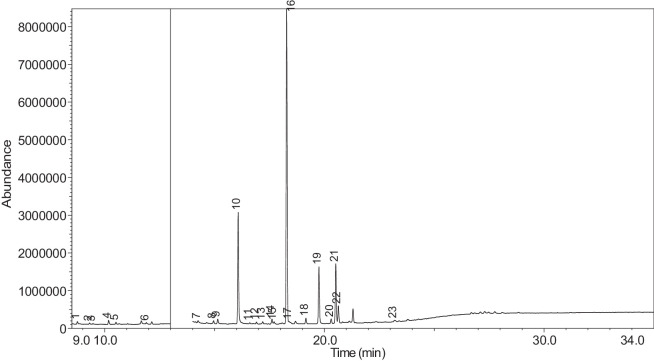
Chromatogram for LV_542_OKT_13. The peak numbers are referred to as follows: 1 = Propylene Glycol; 2 = Methyl propyl ether; 3 = 2-Propanol, 1,3-dichloro-; 4 = 1,2-Propanediol, 1-acetate; 5 = 1,2-Propanediol, 2-acetate; 6 = Methyl propionate; 7 = Glycerin; 8 = Isophorone; 9 = 1,2,3-Propanetriol, 1-acetate; 10 = Ethyl maltol; 11 = Alpha-monopropionin; 12 = 1,3-Dioxolane, 4-methyl-2-phenyl-; 13 = Benzaldehyde, 4-methoxy-; 14 = Benzenemethanol, 4-methoxy-; 15 = 5-Thiazoleethanol, 4-methyl-; 16 = Pyridine, 3-(1-methyl-2-pyrrolidinyl)-, (S)-; 17 = p-Dioxane-2,5-dimethanol; 18 = 2(3H)-Furanone, dihydro-5-pentyl-; 19 = Benzaldehyde, 3-hydroxy-4-methoxy-; 20 = (1′s,2′s)-Nicotine-N'-oxide; 21 = Ethyl Vanillin; 22 = Mandelic acid, 3,4-dimethoxy-, methyl ester; 23 = Vanillin propylene glycol acetal.

**Fig. 45:**
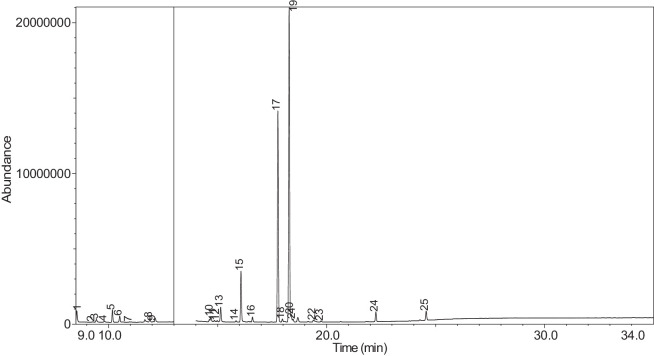
Chromatogram for LV_542_OKT_14. The peak numbers are referred to as follows: 1 = Butanoic acid, 2-methyl-, ethyl ester; 2 = 3-Hexen-1-ol, (Z)-; 3 = 1-Hexanol; 4 = Dimethyl Sulfoxide; 5 = 1,2-Propanediol, 1-acetate; 6 = 1,2-Propanediol, 2-acetate; 7 = 2-(2-Methoxyethoxy)ethyl acetate; 8 = Methyl propionate; 9 = D-Limonene; 10 = 1-Propanol, 2-ethoxy-; 11 = Propanoic acid, 2-hydroxy-, methyl ester, (.±.)-; 12 = 2-Butanol, (R)-; 13 = 1,2,3-Propanetriol, 1-acetate; 14 = 1,2-Ethanediol, diacetate; 15 = Ethyl maltol; 16 = Alpha-monopropionin; 17 = .alpha.-d-Erythro-hex-2-enopyranoside, ethyl 2,3-dideoxy-; 18 = 2,3-dihydroxypropyl isobutyrate; 19 = Pyridine, 3-(1-methyl-2-pyrrolidinyl)-, (S)-; 20 = Pyridine, 3-(1-methyl-2-pyrrolidinyl)-, (S)-; 21 = Methyl anthranilate; 22 = Acetic acid, (2-methoxyethoxy)-; 23 = Benzaldehyde, 3-hydroxy-4-methoxy-; 24 = Cyclohexanecarboxamide, N-ethyl-5-methyl-2-(1-methylethyl)-; 25 = 4-(4-Hydroxyphenyl)-2-butanone propyleneglycol.

**Fig. 46:**
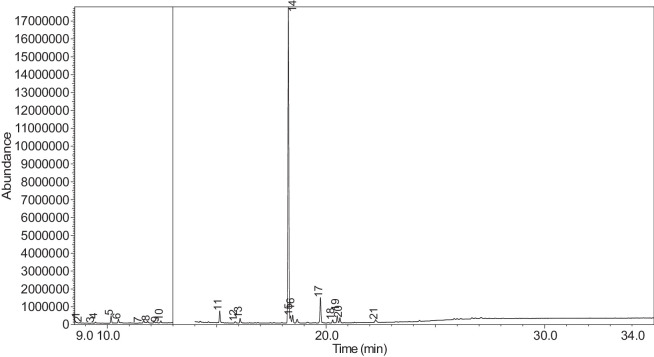
Chromatogram for LV_542_OKT_15. The peak numbers are referred to as follows: 1 = Butanoic acid, 2-methyl-, ethyl ester; 2 = Propylene Glycol; 3 = Methyl propyl ether; 4 = 2-Propanol, 1,3-dichloro-; 5 = 1,2-Propanediol, 1-acetate; 6 = 1,2-Propanediol, 2-acetate; 7 = Butanoic acid, butyl ester; 8 = Dihydroxyacetone; 9 = 2(3H)-Furanone, dihydro-5-methyl-; 10 = Butanoic acid, 3-methyl-, butyl ester; 11 = 1,2,3-Propanetriol, 1-acetate; 12 = 1,2-Epoxy-3-propyl acetate; 13 = Ethyl maltol; 14 = Pyridine, 3-(1-methyl-2-pyrrolidinyl)-, (S)-; 15 = Pyridine, 3-(1-methyl-2-pyrrolidinyl)-, (S)-; 16 = Piperonal; 17 = Benzaldehyde, 3-hydroxy-4-methoxy-; 18 = (1′s,2′s)-Nicotine-N'-oxide; 19 = Ethyl Vanillin; 20 = Benzaldehyde, 3,4-dimethoxy-, methylmonoacetal; 21 = 2H-1-Benzopyran-2-one, 6-methyl-.

**Fig. 47:**
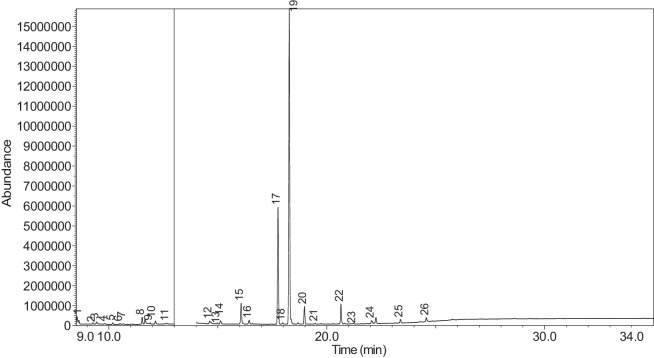
Chromatogram for LV_542_OKT_16. The peak numbers are referred to as follows: 1 = Butanoic acid, 2-methyl-, ethyl ester; 2 = 3-Hexen-1-ol, (Z)-; 3 = 1-Hexanol; 4 = Dimethylsulfoxonium formylmethylide; 5 = 1,2-Propanediol, 1-acetate; 6 = 1,2-Propanediol, 2-acetate; 7 = Cyclotetrasiloxane, octamethyl-; 8 = Hexanoic acid, ethyl ester; 9 = Methyl propionate; 10 = D-Limonene; 11 = Butanoic acid, 3-methylbutyl ester; 12 = Maltol; 13 = 1,3-Dioxolane-2-acetic acid, 2,4-dimethyl-, ethyl ester; 14 = 1,3-Dioxolane-2-acetic acid, 2,4-dimethyl-, ethyl ester; 15 = Ethyl maltol; 16 = Geraniol; 17 = .alpha.-d-Erythro-hex-2-enopyranoside, ethyl 2,3-dideoxy-; 18 = 2,3-dihydroxypropyl isobutyrate; 19 = Pyridine, 3-(1-methyl-2-pyrrolidinyl)-, (S)-; 20 = Cyclohexanepropanoic acid, 2-propenyl ester; 21 = Benzoic acid, 2-amino-, ethyl ester; 22 = 2(3H)-Furanone, 5-hexyldihydro-; 23 = Cyclooctasiloxane, hexadecamethyl-; 24 = 2(3H)-Furanone, 5-heptyldihydro-; 25 = .gamma.-Dodecalactone; 26 = 4-(4-Hydroxyphenyl)-2-butanone propyleneglycol.

**Fig. 48:**
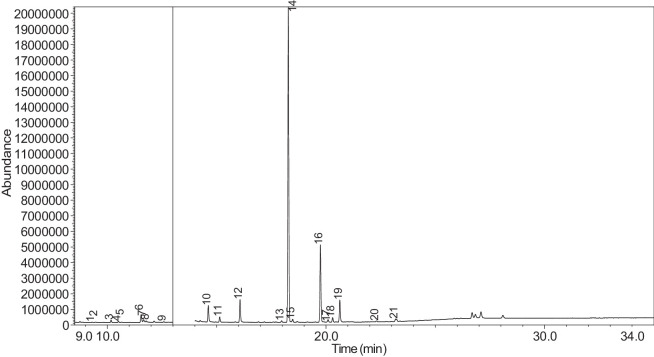
Chromatogram for LV_542_OKT_17. The peak numbers are referred to as follows: 1 = Methyl propyl ether; 2 = 2-Propanol, 1,3-dichloro-; 3 = 1,2-Propanediol, 1-acetate; 4 = 1,2-Propanediol, 2-acetate; 5 = Cyclotetrasiloxane, octamethyl-; 6 = 1,3-Dioxolane, 2,2,4-trimethyl-; 7 = Silane, triethylmethoxy-; 8 = Pyrazine, trimethyl-; 9 = Acetylpyrazine; 10 = Maltol; 11 = 1,2,3-Propanetriol, 1-acetate; 12 = Ethyl maltol; 13 = 2,3-dihydroxypropyl isobutyrate; 14 = Pyridine, 3-(1-methyl-2-pyrrolidinyl)-, (S)-; 15 = Piperonal; 16 = Benzaldehyde, 3-hydroxy-4-methoxy-; 17 = 1,4-Benzenediol, 2-methoxy-; 18 = (1′s,2′s)-Nicotine-N'-oxide; 19 = Mandelic acid, 3,4-dimethoxy-, methyl ester; 20 = 1,3-Benzodioxole, 5-(4-methyl-1,3-dioxolan-2-yl)-; 21 = Vanillin propylene glycol acetal.

**Fig. 49:**
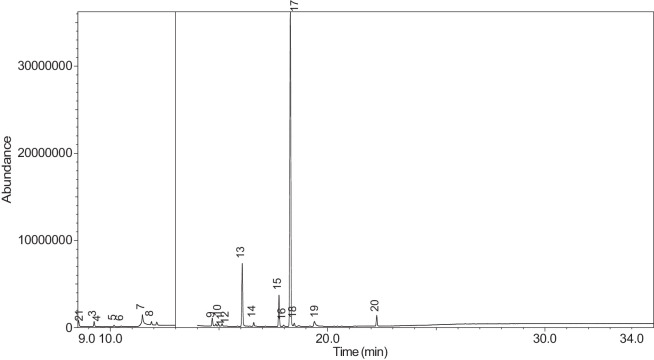
Chromatogram for LV_542_OKT_18. The peak numbers are referred to as follows:1 = Butanoic acid, 2-methyl-, ethyl ester; 2 = 2,3-Butanediol, [R-(R*,R*)]-; 3 = 3-Hexen-1-ol, (Z)-; 4 = 2-Propanol, 1,3-dichloro-; 5 = 1,2-Propanediol, 1-acetate; 6 = 1,2-Propanediol, 2-acetate; 7 = Tetraethylene glycol; 8 = Methyl propionate; 9 = 1-Propanol, 2-ethoxy-; 10 = 2-Butanol, (R)-; 11 = 2-Butanol, (R)-; 12 = 1,2,3-Propanetriol, 1-acetate; 13 = Ethyl maltol; 14 = Alpha-monopropionin; 15 = .alpha.-d-Erythro-hex-2-enopyranoside, ethyl 2,3-dideoxy-; 16 = 2,3-dihydroxypropyl isobutyrate; 17 = Pyridine, 3-(1-methyl-2-pyrrolidinyl)-, (S)-; 18 = Methyl anthranilate; 19 = Acetic acid, (2-methoxyethoxy)-; 20 = Cyclohexanecarboxamide, N-ethyl-5-methyl-2-(1-methylethyl)-.

**Fig. 50:**
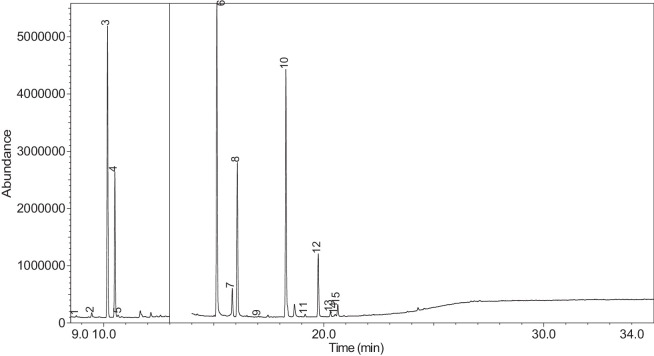
Chromatogram for LV_542_OKT_19. The peak numbers are referred to as follows: 1 = Propylene Glycol; 2 = 2-Propanol, 1,3-dichloro-; 3 = 1,2-Propanediol, 1-acetate; 4 = 1,2-Propanediol, 2-acetate; 5 = Cyclotetrasiloxane, octamethyl-; 6 = 1,2,3-Propanetriol, 1-acetate; 7 = 1,2,3-Propanetriol, 1-acetate; 8 = Ethyl maltol; 9 = 1,2,3-Propanetriol, 1-acetate; 10 = Pyridine, 3-(1-methyl-2-pyrrolidinyl)-, (S)-; 11 = 2(3H)-Furanone, dihydro-5-pentyl-; 12 = Benzaldehyde, 3-hydroxy-4-methoxy-; 13 = (1′s,2′s)-Nicotine-N'-oxide; 14 = Ethyl Vanillin; 15 = Mandelic acid, 3,4-dimethoxy-, methyl ester.

**Fig. 51:**
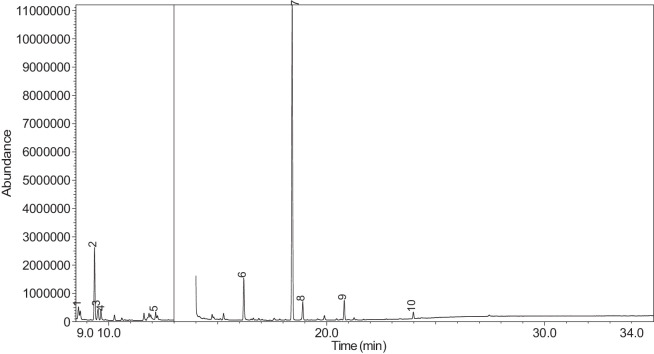
Chromatogram for LV_542_OKT_20. The peak numbers are referred to as follows:1 = Butanoic acid, 2-methyl-, ethyl ester; 2 = 3-Hexen-1-ol, (Z)-; 3 = 1-Hexanol; 4 = Butanoic acid, 2-methyl-; 5 = Hexanoic acid; 6 = Ethyl maltol; 7 = Pyridine, 3-(1-methyl-2-pyrrolidinyl)-, (S)-; 8 = 2-Propenoic acid, 3-phenyl-, methyl ester; 9 = 2(3H)-Furanone, 5-hexyldihydro-; 10 = .delta.-Dodecalactone.

**Fig. 52:**
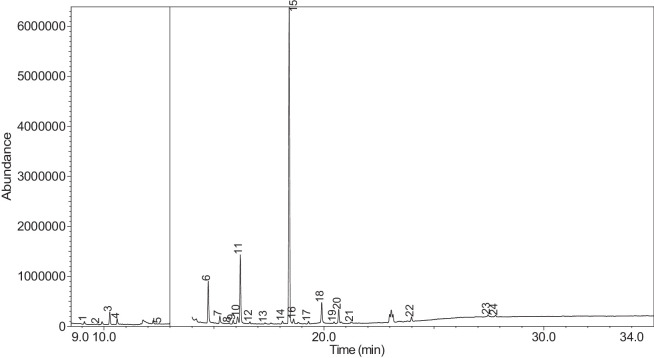
Chromatogram for LV_542_OKT_21. The peak numbers are referred to as follows:1 = Furfural; 2 = 2-Furanmethanol; 3 = 1,2-Propanediol, 1-acetate; 4 = 1,2-Propanediol, 2-acetate; 5 = 1,2-Propanediol, diacetate; 6 = Maltol; 7 = 1,2,3-Propanetriol, 1-acetate; 8 = 2-Acetoxyisobutyryl chloride; 9 = 2-Acetoxyisobutyryl chloride; 10 = 2-Acetoxyisobutyryl chloride; 11 = Ethyl maltol; 12 = 4-Methyl-2-oxopentanenitrile; 13 = Benzaldehyde, 4-methoxy-; 14 = 2,3-dihydroxypropyl isobutyrate; 15 = Pyridine, 3-(1-methyl-2-pyrrolidinyl)-, (S)-; 16 = Piperonal; 17 = 2(3H)-Furanone, dihydro-5-pentyl-; 18 = Vanillin; 19 = (1′s,2′s)-Nicotine-N'-oxide; 20 = Ethyl Vanillin; 21 = 1,2,3,6-Tetrahydro-2,3′-bipyridine; 22 = .delta.-Dodecalactone; 23 = n-Nonadecanol-1; 24 = Methyl stearate.

**Fig. 53:**
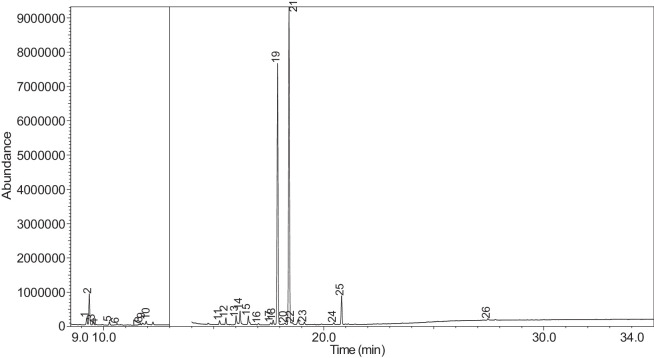
Chromatogram for LV_542_OKT_22. The peak numbers are referred to as follows:1 = 1-Butanol, 3-methyl-, acetate; 2 = 3-Hexen-1-ol, (Z)-; 3 = 1-Hexanol; 4 = Butanoic acid, 2-methyl-; 5 = 1,2-Propanediol, 1-acetate; 6 = 1,2-Propanediol, 2-acetate; 7 = Hexanoic acid, ethyl ester; 8 = Benzaldehyde; 9 = 1-Dimethyl(octyl)silyloxypropane; 10 = Acetic acid, hexyl ester; 11 = 1,2,3-Propanetriol, 1-acetate; 12 = Benzenemethanol, .alpha.-methyl-, acetate; 13 = Benzoic acid; 14 = Ethyl maltol; 15 = Geraniol; 16 = 1,3-Dioxolane, 4-methyl-2-phenyl-; 17 = Dianhydromannitol; 18 = 2(3H)-Furanone, 5-butyldihydro-; 19 = .alpha.-d-Erythro-hex-2-enopyranoside, ethyl 2,3-dideoxy-; 20 = 1,4-Dioxane-2,6-dimethanol; 21 = Pyridine,3-(1-methyl-2-pyrrolidinyl)-,(S)-; 22 = p-Dioxane-2,5-dimethanol; 3 = Cyclohexanepropanoic acid, 2-propenyl ester; 24 = (1′s,2′s)-Nicotine-N'-oxide; 25 = 2(3H)-Furanone, 5-hexyldihydro-; 26 = 1-Octadecanol.

**Fig. 54:**
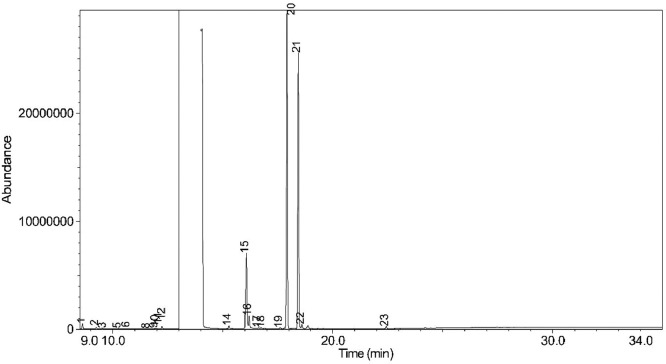
Chromatogram for LV_542_OKT_23. The peak numbers are referred to as follows:1 = Butanoic acid, 2-methyl-, ethyl ester; 2 = 1-Butanol, 3-methyl-, acetate; 3 = 1-Hexanol; 4 = 2-Propanol, 1,3-dichloro-; 5 = 1,2-Propanediol, 1-acetate; 6 = 1,2-Propanediol, 2-acetate; 7 = 1,3-Dioxolane-2-methanol, 2,4-dimethyl-; 8 = utanoic acid, butyl ester; 9 = Acetic acid, hexyl ester; 10 = Methyl propionate; 11 = Glycolaldehyde dimethyl acetal; 12 = D-Limonene; 13 = Glycerin; 14 = 1,2,3-Propanetriol, 1-acetate; 15 = Benzoic acid; 16 = Ethyl maltol; 17 = 4-Methyl-2-oxopentanenitrile; 18 = Alpha-monopropionin; 19 = Isosorbide; 20 = .alpha.-d-Erythro-hex-2-enopyranoside, ethyl 2,3-dideoxy-; 21 = Pyridine, 3-(1-methyl-2-pyrrolidinyl)-, (S)-; 22 = Methyl anthranilate; 23 = Cyclohexanecarboxamide,N-ethyl-5-methyl-2-(1-methylethyl)-.

**Fig. 55:**
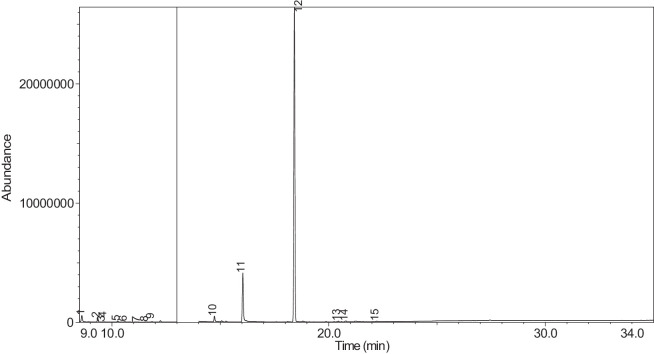
Chromatogram for LV_542_OKT_24. The peak numbers are referred to as follows:1 = Butanoic acid, 2-methyl-, ethyl ester; 2 = 3-Hexen-1-ol, (Z)-; 3 = 2-Propanol, 1,3-dichloro-; 4 = Butanoic acid, 2-methyl-; 5 = 1,2-Propanediol, 1-acetate; 6 = 1,2-Propanediol, 2-acetate; 7 = Decane; 8 = Butanoic acid, butyl ester; 9 = 3-Hexen-1-ol, acetate, (Z)-; 10 = Maltol; 11 = Benzoic acid; 12 = Pyridine, 3-(1-methyl-2-pyrrolidinyl)-, (S)-; 13 = (1′s,2′s)-Nicotine-N'-oxide; 14 = 2(3H)-Furanone, 5-hexyldihydro-; 15 = 2(3H)-Furanone, 5-heptyldihydro-.

**Fig. 56:**
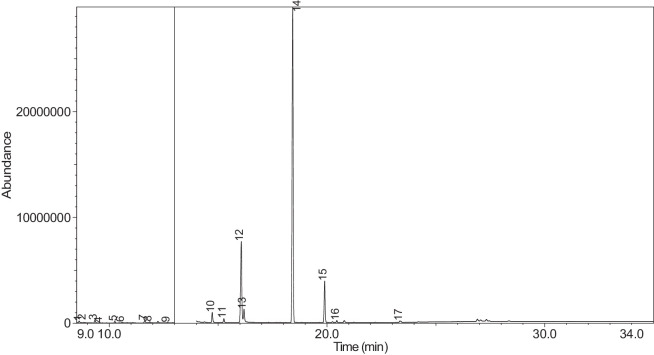
Chromatogram for LV_542_OKT_25. The peak numbers are referred to as follows:1 = Butanoic acid, 2-methyl-, ethyl ester; 2 = Propylene Glycol; 3 = 3-Hexen-1-ol, (Z)-; 4 = Propanoic acid, chloro-2-hydroxy-; 5 = 1,2-Propanediol, 1-acetate; 6 = 1,2-Propanediol, 2-acetate; 7 = 1,3-Dioxolane, 2,2,4-trimethyl-; 8 = Pyrazine, trimethyl-; 9 = Acetylpyrazine; 10 = Maltol; 11 = 1,2,3-Propanetriol, 1-acetate; 12 = Benzoic acid; 13 = Ethyl maltol; 14 = Pyridine, 3-(1-methyl-2-pyrrolidinyl)-, (S)-; 15 = Vanillin; 16 = (1′s,2′s)-Nicotine-N'-oxide; 17 = Vanillin propylene glycol acetal.

**Fig. 57:**
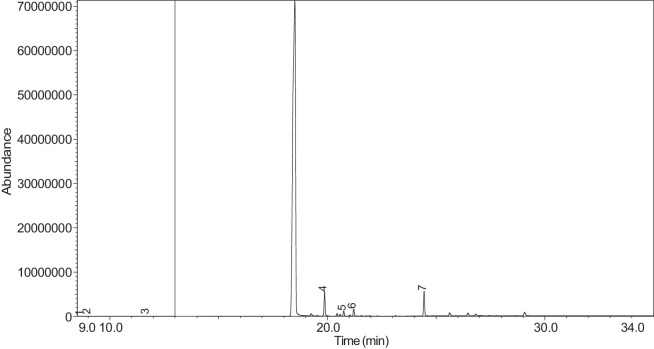
Chromatogram for NIC_IPIN_MAY. The peak numbers are referred to as follows:1 = Formamide, N-methyl-; 2 = Ethylbenzene; 3 = Octane, 3,3-dimethyl-; 4 = Pyridine, 3-(3,4-dihydro-2H-pyrrol-5-yl)-; 5 = Anabasine; 6 = 1,2,3,6-Tetrahydro-2,3′-bipyridine; 7 = Cotinine.

Table 4 presents the results of GC-MS analysis performed on the 27 samples including peak number, retention time, identified components, mass-to-charge ratio (m/z) value, chemical abstracts service (CAS) registry number, and the percentage area of the compound detected.

**Table 4:** Chemical compounds detected in the GC-MS analysis of all samples.

No.	Lot number	Peak	RT^1^(min)	Component	m/z value	CAS number	Area (%)
1	LV_542_OKT_1	1	8.744	Propylene Glycol	45.00	57-55-6	0.73
		2	9.3	Methyl propyl ether	45.00	557-17-5	0.3
		3	9.453	2-Propanol, 1,3-dichloro-	79.00	96-23-1	0.17
		4	10.171	1,2-Propanediol, 1-acetate	43.00	627-69-0	0.34
		5	10.506	1,2-Propanediol, 2-acetate	43.00	03/01/6214	0.21
		6	10.657	Cyclotetrasiloxane, octamethyl-	281.00	556-67-2	0.31
		7	11.041	Oxime-, methoxy-phenyl-	133.00	67160-14-9	0.17
		8	11.545	1,3-Dioxolane, 2,2,4-trimethyl-	43.00	1193-11-9	0.86
		9	11.894	Methyl propionate	57.00	554-12-1	0.18
		10	14.036	Phenol, 2-methoxy-	109.00	90-05-1	0.19
		11	14.135	2(3H)-Furanone, 5-ethyldihydro-	85.00	695-06-7	0.52
		12	14.246	Glycerin	61.00	56-81-5	0.82
		13	14.631	Maltol	126.00	118-71-8	0.13
		14	15.042	Benzene, 1,4-dimethoxy-	123.00	150-78-7	0.29
		15	15.144	1,2,3-Propanetriol, 1-acetate	43.00	106-61-6	1.27
		16	16.071	Ethyl maltol	140.00	08/11/4940	10.23
		17	16.6	Alpha-monopropionin	57.00	624-47-5	0.64
		18	17.188	Benzaldehyde, 4-methoxy-	135.00	123-11-5	0.54
		19	17.61	Benzenemethanol, 4-methoxy-	109.00	105-13-5	0.31
		20	17.707	5-Thiazoleethanol, 4-methyl-	112.00	137-00-8	1.37
		21	17.973	2,3-dihydroxypropyl isobutyrate	71.00	557-25-5	0.17
		22	18.092	1,4-Dioxane-2,6-dimethanol	57.00	54120-69-3	0.29
		23	18.278	Pyridine, 3-(1-methyl-2-pyrrolidinyl)-, (S)-	84.00	54-11-5	24.52
		24	18.424	p-Dioxane-2,5-dimethanol	57.00	14236-12-5	0.46
		25	19.154	2(3H)-Furanone, dihydro-5-pentyl-	85.00	104-61-0	1.34
		26	19.747	Benzaldehyde, 3-hydroxy-4-methoxy-	151.00	621-59-0	12.12
		27	20.098	1,4-Benzenediol, 2-methoxy-	140.00	824-46-4	0.12
		28	20.515	Ethyl Vanillin	137.00	121-32-4	11.61
		29	20.634	Mandelic acid, 3,4-dimethoxy-, methyl ester	167.00	2911-73-1	4.64
		30	21.101	2H-Pyran-2-one, tetrahydro-6-pentyl-	99.00	705-86-2	0.19
		31	23.183	Vanillin propylene glycol acetal	151.00	68527-74-2	0.09
		32	23.802	.delta.-Dodecalactone	99.00	713-95-1	0.29
2	LV_542_OKT_2	1	8.539	Butanoic acid, 2-methyl-, ethyl ester	57.00	7452-79-1	0.74
		2	8.889	Ethanedioic acid, dimethyl ester	59.00	553-90-2	0.17
		3	9.257	3-Hexen-1-ol, (Z)-	41.00	928-96-1	1.28
		4	9.445	1-Hexanol	43.00	111-27-3	0.51
		5	9.554	Butanoic acid, 2-methyl-	74.00	116-53-0	0.14
		6	9.895	Acetic acid, pentyl ester	43.00	628-63-7	0.13
		7	10.171	1,2-Propanediol, 1-acetate	43.00	627-69-0	1.94
		8	10.507	1,2-Propanediol, 2-acetate	43.00	03/01/6214	1.21
		9	10.857	1,3-Dioxolane-2-methanol, 2,4-dimethyl-	43.00	53951-43-2	0.09
		10	11.528	Hexanoic acid, ethyl ester	88.00	123-66-0	0.05
		11	11.893	Methyl propionate	57.00	554-12-1	0.97
		12	12.634	Butanoic acid, 3-methylbutyl ester	71.00	106-27-4	0.12
		13	15.146	1,2,3-Propanetriol, 1-acetate	43.00	106-61-6	9.04
		14	15.851	1,2-Ethanediol, diacetate	43.00	111-55-7	0.67
		15	16.074	Ethyl maltol	140.00	08/11/4940	13.67
		16	16.6	Alpha-monopropionin	57.00	624-47-5	5.04
		17	17.767	.alpha.-d-Erythro-hex-2-enopyranoside, ethyl 2,3-dideoxy-	114.00	23339-15-3	41.91
		18	17.974	2,3-dihydroxypropyl isobutyrate	71.00	557-25-5	0.63
		19	18.28	Pyridine, 3-(1-methyl-2-pyrrolidinyl)-, (S)-	84.00	54-11-5	13
		20	18.466	Methyl anthranilate	119.00	134-20-3	4.81
		21	19.183	2-Buten-1-one, 1-(2,6,6-trimethyl-1-cyclohexen-1-yl)-	177.00	23726-92-3	0.23
		22	20.655	2(3H)-Furanone, 5-hexyldihydro-	85.00	706-14-9	1.07
		23	22.16	p-Dioxane-2,5-dimethanol	57.00	14236-12-5	0.89
		24	22.261	Cyclohexanecarboxamide, N-ethyl-5-methyl-2-(1-methylethyl)-	87.00	68489-00-9	1.55
		25	22.734	1,4-Dioxane-2,6-dimethanol	57.00	54120-69-3	0.14
3	LV_542_OKT_3	1	8.538	Butanoic acid, 2-methyl-, ethyl ester	57.00	7452-79-1	0.93
		2	9.255	3-Hexen-1-ol, (Z)-	41.00	928-96-1	5.06
		3	9.454	2-Propanol, 1,3-dichloro-	79.00	96-23-1	1.04
		4	9.551	Butanoic acid, 2-methyl-	74.00	116-53-0	0.15
		5	9.895	Acetic acid, pentyl ester	43.00	628-63-7	0.32
		6	10.171	1,2-Propanediol, 1-acetate	43.00	627-69-0	2.51
		7	10.507	1,2-Propanediol, 2-acetate	43.00	03/01/6214	1.57
		8	10.656	Cyclotetrasiloxane, octamethyl-	281.00	556-67-2	0.43
		9	10.856	1,3-Dioxolane-2-methanol, 2,4-dimethyl-	43.00	53951-43-2	0.24
		10	11.177	(R)-(-)-2,2-Dimethyl-1,3-dioxolane-4-methanol	43.00	14347-78-5	0.24
		11	11.529	Hexanoic acid, ethyl ester	88.00	123-66-0	0.16
		12	12.044	D-Limonene	68.00	5989-27-5	0.28
		13	12.297	Eucalyptol	43.00	470-82-6	0.12
		14	12.635	Butanoic acid, 3-methylbutyl ester	71.00	106-27-4	0.47
		15	14.88	Cyclohexanol, 1-methyl-4-(1-methylethenyl)-	43.00	138-87-4	0.09
		16	15.081	Octanoic acid, ethyl ester	88.00	106-32-1	0.14
		17	15.144	1,2,3-Propanetriol, 1-acetate	43.00	106-61-6	3.11
		18	15.31	3-Cyclohexen-1-ol, 4-methyl-1-(1-methylethyl)-, (R)-	71.00	20126-76-5	0.12
		19	15.639	L-.alpha.-Terpineol	59.00	10482-56-1	2.59
		20	15.715	Cyclohexanol, 1-methyl-4-(1-methylethylidene)-	121.00	586-81-2	0.22
		21	16.087	2,6-Octadien-1-ol, 3,7-dimethyl-, (Z)-	69.00	106-25-2	0.24
		22	16.464	1,3-Dioxolane, 2-heptyl-4-methyl-	87.00	74094-61-4	0.37
		23	16.689	(-)-Carvone	82.00	6485-40-1	0.79
		24	17.042	1,2,3-Propanetriol, 1-acetate	43.00	106-61-6	0.76
		25	17.765	.alpha.-d-Erythro-hex-2-enopyranoside, ethyl 2,3-dideoxy-	114.00	23339-15-3	49.89
		26	18.059	Triacetin	43.00	102-76-1	1.06
		27	18.28	Pyridine, 3-(1-methyl-2-pyrrolidinyl)-, (S)-	84.00	54-11-5	16.76
		28	18.407	Pyridine, 3-(1-methyl-2-pyrrolidinyl)-, (S)-	84.00	54-11-5	2.86
		29	19.475	.alpha.-Ionone	121.00	127-41-3	0.21
		30	19.751	Benzaldehyde, 3-hydroxy-4-methoxy-	151.00	621-59-0	1.49
		31	20.146	Butylated Hydroxytoluene	205.00	128-37-0	0.15
		32	20.278	trans-.beta.-Ionone	177.00	79-77-6	0.37
		33	20.518	Ethyl Vanillin	137.00	121-32-4	1.56
		34	20.655	2(3H)-Furanone, 5-hexyldihydro-	85.00	706-14-9	1.24
		35	22.159	p-Dioxane-2,5-dimethanol	57.00	14236-12-5	1.61
		36	22.262	Cyclohexanecarboxamide, N-ethyl-5-methyl-2-(1-methylethyl)-	100.00	68489-00-9	0.51
		37	22.732	p-Dioxane-2,5-dimethanol	57.00	14236-12-5	0.33
4	LV_542_OKT_4	1	8.756	Propylene Glycol	45.00	57-55-6	0.21
		2	9.463	2-Propanol, 1,3-dichloro-	79.00	96-23-1	0.14
		3	10.023	2-Furanmethanol, tetrahydro-	71.00	97-99-4	0.52
		4	10.176	1,2-Propanediol, 1-acetate	43.00	627-69-0	2.76
		5	10.511	1,2-Propanediol, 2-acetate	43.00	03/01/6214	1.69
		6	10.66	Cyclotetrasiloxane, octamethyl-	281.00	556-67-2	0.26
		7	10.807	1,2-Propanediol, 3-methoxy-	45.00	623-39-2	0.39
		8	11.898	Methyl propionate	57.00	554-12-1	0.16
		9	15.141	1,2,3-Propanetriol, 1-acetate	43.00	106-61-6	3.9
		10	16.076	Ethyl maltol	140.00	08/11/4940	0.78
		11	17.574	2(3H)-Furanone, 5-butyldihydro-	85.00	104-50-7	0.59
		12	18.078	1,4-Dioxane-2,6-dimethanol	57.00	54120-69-3	0.34
		13	18.282	Pyridine, 3-(1-methyl-2-pyrrolidinyl)-, (S)-	84.00	54-11-5	67.6
		14	18.402	p-Dioxane-2,5-dimethanol	57.00	14236-12-5	0.47
		15	19.158	2(3H)-Furanone, dihydro-5-pentyl-	85.00	104-61-0	0.28
		16	19.751	Benzaldehyde, 3-hydroxy-4-methoxy-	151.00	621-59-0	4.69
		17	20.309	(1′s,2′s)-Nicotine-N'-oxide	119.00	51095-86-4	0.47
		18	20.52	Ethyl Vanillin	137.00	121-32-4	11.72
		19	20.637	Mandelic acid, 3,4-dimethoxy-, methyl ester	167.00	2911-73-1	1.45
		20	21.304	Danielone	181.00	90426-22-5	1.57
5	LV_542_OKT_5A	1	9.268	3-Hexen-1-ol, (Z)-	41.00	928-96-1	0.18
		2	9.466	2-Propanol, 1,3-dichloro-	79.00	96-23-1	0.47
		3	10.181	1,2-Propanediol, 1-acetate	43.00	627-69-0	0.85
		4	10.516	1,2-Propanediol, 2-acetate	43.00	03/01/6214	0.42
		5	11.051	Oxime-, methoxy-phenyl-	133.00	67160-14-9	0.09
		6	15.15	1,2,3-Propanetriol, 1-acetate	43.00	106-61-6	1.16
		7	17.776	.alpha.-d-Erythro-hex-2-enopyranoside, ethyl 2,3-dideoxy-	114.00	23339-15-3	73.2
		8	18.288	Pyridine, 3-(1-methyl-2-pyrrolidinyl)-, (S)-	84.00	54-11-5	17.68
		9	18.38	Pyridine, 3-(1-methyl-2-pyrrolidinyl)-, (S)-	84.00	54-11-5	1.92
		10	20.662	2(3H)-Furanone, 5-hexyldihydro-	85.00	706-14-9	0.8
		11	21.11	2H-Pyran-2-one, tetrahydro-6-pentyl-	99.00	705-86-2	0.32
		12	22.069	2(3H)-Furanone, 5-heptyldihydro-	85.00	104-67-6	2.89
6	LV_542_OKT_5B	1	9.471	2-Propanol, 1,3-dichloro-	79.00	96-23-1	0.15
		2	17.787	Hexanamide, 3,5,5-trimethyl-N-pentyl-	114.00	1700656-13-8	99.85
7	LV_542_OKT_6	1	8.632	Butanoic acid, 3-methyl-, ethyl ester	88.00	108-64-5	1.88
		2	8.772	Propylene Glycol	45.00	57-55-6	0.26
		3	9.164	1-Butanol, 3-methyl-, acetate	43.00	123-92-2	36.28
		4	9.444	1-Hexanol	56.00	111-27-3	17.53
		5	10.083	3-Methoxyhex-1-ene	71.00	3404-61-3	0.15
		6	10.184	1,2-Propanediol, 1-acetate	43.00	627-69-0	2.46
		7	10.519	1,2-Propanediol, 2-acetate	43.00	03/01/6214	1.64
		8	10.665	Cyclotetrasiloxane, octamethyl-	281.00	556-67-2	0.2
		9	10.87	1,3-Dioxolane-2-methanol, 2,4-dimethyl-	43.00	53951-43-2	0.16
		10	10.934	1,3-Dioxolane-2-methanol, 2,4-dimethyl-	43.00	53951-43-2	0.09
		11	11.163	trans-2-Hexenal dimethyl acetal	71.00	18318-83-7	0.39
		12	11.538	Hexanoic acid, ethyl ester	88.00	123-66-0	0.22
		13	11.77	3-Hexen-1-ol, acetate, (E)-	43.00	3681-82-1	0.09
		14	11.846	Acetic acid, hexyl ester	43.00	142-92-7	1.67
		15	15.029	Butanoic acid, hexyl ester	43.00	2639-63-6	0.16
		16	15.148	1,2,3-Propanetriol, 1-acetate	43.00	106-61-6	3.18
		17	17.2	3-Pentanol, 2,3,4-trimethyl-	87.00	3054-92-0	0.81
		18	17.773	.alpha.-d-Erythro-hex-2-enopyranoside, ethyl 2,3-dideoxy-	114.00	23339-15-3	23.75
		19	18.29	Pyridine, 3-(1-methyl-2-pyrrolidinyl)-, (S)-	84.00	54-11-5	7.8
		20	18.367	Pyridine, 3-(1-methyl-2-pyrrolidinyl)-, (S)-	84.00	54-11-5	1.09
		21	20.317	(1′s,2′s)-Nicotine-N'-oxide	119.00	51095-86-4	0.17
8	LV_542_OKT_7	1	8.676	1,3-Dioxolane-2-methanol, 2,4-dimethyl-	43.00	53951-43-2	0.1
		2	9.162	1-Butanol, 3-methyl-, acetate	43.00	123-92-2	2.56
		3	9.267	3-Hexen-1-ol, (E)-	67.00	928-97-2	0.34
		4	9.468	2-Propanol, 1,3-dichloro-	79.00	96-23-1	0.48
		5	9.609	2-Furanmethanol	98.00	98-00-0	0.12
		6	9.813	Dimethyl Sulfoxide	63.00	67-68-5	0.27
		7	10.181	1,2-Propanediol, 1-acetate	43.00	627-69-0	10.43
		8	10.516	1,2-Propanediol, 2-acetate	43.00	03/01/6214	5.98
		9	10.867	1,3-Dioxolane-2-methanol, 2,4-dimethyl-	43.00	53951-43-2	0.12
		10	11.493	Butanoic acid, butyl ester	71.00	109-21-7	1.16
		11	11.704	2-Furanmethanol, acetate	81.00	623-17-6	1.52
		12	11.846	Acetic acid, hexyl ester	43.00	142-92-7	1.08
		13	12.054	D-Limonene	67.00	5989-27-5	0.26
		14	12.284	1,3,6-Octatriene, 3,7-dimethyl-, (Z)-	93.00	3338-55-4	0.03
		15	12.422	1,2-Propanediol, diacetate	43.00	623-84-7	0.21
		16	12.643	Butanoic acid, 3-methylbutyl ester	71.00	106-27-4	0.58
		17	14.837	Allyl heptanoate	43.00	142-19-8	2.12
		18	15.152	1,2,3-Propanetriol, 1-acetate	43.00	106-61-6	11.95
		19	15.447	Benzenemethanol, .alpha.-methyl-, acetate	122.00	93-92-5	1.03
		20	15.858	1,2,3-Propanetriol, 1-acetate	43.00	106-61-6	1.27
		21	16.083	Ethyl maltol	140.00	08/11/4940	9.79
		22	16.521	2,3-Butanedione, monooxime	43.00	57-71-6	0.18
		23	17.052	1,2,3-Propanetriol, 1-acetate	43.00	106-61-6	0.34
		24	17.143	Glycerol 1,2-diacetate	43.00	102-62-5	0.18
		25	17.582	2(3H)-Furanone, 5-butyldihydro-	85.00	104-50-7	5.21
		26	17.776	.alpha.-d-Erythro-hex-2-enopyranoside, ethyl 2,3-dideoxy-	114.00	23339-15-3	26.32
		27	17.986	2,3-dihydroxypropyl isobutyrate	71.00	557-25-5	0.11
		28	18.295	Pyridine, 3-(1-methyl-2-pyrrolidinyl)-, (S)-	84.00	54-11-5	4.73
		29	18.397	Pyridine, 3-(1-methyl-2-pyrrolidinyl)-, (S)-	84.00	54-11-5	3.43
		30	18.901	Diphenyl ether	170.00	101-84-8	0.36
		31	18.99	Cyclohexanepropanoic acid, 2-propenyl ester	55.00	2705-87-5	1.06
		32	20.667	2(3H)-Furanone, 5-hexyldihydro-	85.00	706-14-9	6.58
		33	23.41	Glycerin, 1,2-dicaprylate	57.00	1069-87-0	0.11
9	LV_542_OKT_8	1	9.47	2-Propanol, 1,3-dichloro-	79.00	96-23-1	0.37
		2	10.186	1,2-Propanediol, 1-acetate	43.00	627-69-0	0.16
		3	10.52	1,2-Propanediol, 2-acetate	43.00	03/01/6214	0.09
		4	11.053	Oxime-, methoxy-phenyl-	133.00	67160-14-9	0.05
		5	11.133	Bicyclo[3.1.1]heptane, 6,6-dimethyl-2-methylene-, (1S)-	93.00	18172-67-3	0.09
		6	11.876	7-Oxabicyclo[2.2.1]heptane, 1-methyl-4-(1-methylethyl)-	43.00	470-67-7	0.16
		7	12.051	D-Limonene	67.00	5989-27-5	0.13
		8	12.305	Eucalyptol	43.00	470-82-6	0.11
		9	15.648	L-.alpha.-Terpineol	59.00	10482-56-1	0.79
		10	15.724	Cyclohexanol, 1-methyl-4-(1-methylethylidene)-	121.00	586-81-2	0.19
		11	17.781	.alpha.-d-Erythro-hex-2-enopyranoside, ethyl 2,3-dideoxy-	114.00	23339-15-3	53.44
		12	18.291	Pyridine, 3-(1-methyl-2-pyrrolidinyl)-, (S)-	84.00	54-11-5	41.91
		13	18.42	Pyridine, 3-(1-methyl-2-pyrrolidinyl)-, (S)-	84.00	54-11-5	2.33
		14	20.316	(1′s,2′s)-Nicotine-N'-oxide	119.00	51095-86-4	0.17
10	LV_542_OKT_9	1	8.628	Butanoic acid, 3-methyl-, ethyl ester	88.00	108-64-5	1.95
		2	9.161	1-Butanol, 3-methyl-, acetate	43.00	123-92-2	2.43
		3	9.465	2-Propanol, 1,3-dichloro-	79.00	96-23-1	0.53
		4	10.181	1,2-Propanediol, 1-acetate	43.00	627-69-0	0.98
		5	10.516	1,2-Propanediol, 2-acetate	43.00	03/01/6214	0.51
		6	11.537	Hexanoic acid, ethyl ester	88.00	123-66-0	0.53
		7	12.454	Butanoic acid, 3-methyl-, butyl ester	85.00	106-27-4	1.89
		8	15.151	1,2,3-Propanetriol, 1-acetate	43.00	106-61-6	2.28
		9	17.777	.alpha.-d-Erythro-hex-2-enopyranoside, ethyl 2,3-dideoxy-	114.00	23339-15-3	56.51
		10	17.982	2,3-dihydroxypropyl isobutyrate	71.00	557-25-5	0.26
		11	18.289	Pyridine, 3-(1-methyl-2-pyrrolidinyl)-, (S)-	84.00	54-11-5	16.26
		12	18.395	Pyridine, 3-(1-methyl-2-pyrrolidinyl)-, (S)-	84.00	54-11-5	1.89
		13	18.476	Methyl anthranilate	119.00	134-20-3	1.07
		14	18.773	2-Propenoic acid, 3-phenyl-, methyl ester	131.00	1754-62-7	0.99
		15	19.483	.alpha.-Ionone	121.00	127-41-3	0.29
		16	19.758	Benzaldehyde, 3-hydroxy-4-methoxy-	152.00	621-59-0	1.19
		17	20.288	3-Buten-2-one, 4-(2,6,6-trimethyl-1-cyclohexen-1-yl)-	177.00	35031-06-2	0.27
		18	20.392	2-Propenoic acid, 3-phenyl-, 1-methylethyl ester	131.00	134990756	3.74
		19	20.526	Ethyl Vanillin	137.00	121-32-4	1.95
		20	20.828	Pentanoic acid, 4-oxo-, butyl ester	99.00	2052-15-5	0.14
		21	20.955	Naphthalene, 2-ethoxy-	144.00	93-18-5	1.74
		22	22.071	2(3H)-Furanone, 5-heptyldihydro-	85.00	104-67-6	2.6
11	LV_542_OKT_10	1	8.544	Butanoic acid, 2-methyl-, ethyl ester	57.00	7452-79-1	0.06
		2	8.895	Ethanedioic acid, dimethyl ester	59.00	553-90-2	0.1
		3	9.264	3-Hexen-1-ol, (Z)-	41.00	928-96-1	0.15
		4	9.463	2-Propanol, 1,3-dichloro-	79.00	96-23-1	0.39
		5	10.179	1,2-Propanediol, 1-acetate	43.00	627-69-0	0.32
		6	10.514	1,2-Propanediol, 2-acetate	43.00	03/01/6214	0.17
		7	12.05	D-Limonene	67.00	5989-27-5	0.03
		8	12.915	Phenol	94.00	108-95-2	0.27
		9	15.149	1,2,3-Propanetriol, 1-acetate	43.00	106-61-6	0.61
		10	15.584	Benzene, 1-methoxy-4-propyl-	121.00	104-45-0	10.09
		11	17.055	Anethole	148.00	104-46-1	2.1
		12	17.474	(Z)-3-Phenylacrylaldehyde	131.00	57194-69-1	0.47
		13	17.773	.alpha.-d-Erythro-hex-2-enopyranoside, ethyl 2,3-dideoxy-	114.00	23339-15-3	44.17
		14	17.979	2,3-dihydroxypropyl isobutyrate	71.00	557-25-5	0.22
		15	18.286	Pyridine, 3-(1-methyl-2-pyrrolidinyl)-, (S)-	84.00	54-11-5	35.41
		16	18.385	Pyridine, 3-(1-methyl-2-pyrrolidinyl)-, (S)-	84.00	54-11-5	1.71
		17	19.756	Benzaldehyde, 3-hydroxy-4-methoxy-	151.00	621-59-0	0.14
		18	21.87	Benzoic acid, 2-hydroxy-, 2-propenyl ester	120.00	10484-09-0	1.74
		19	22.031	Benzoic acid, 2-hydroxy-, 1-methylethyl ester	120.00	607-85-2	0.99
		20	25.909	Benzoic acid, 2-hydroxy-, 2-methylpropyl ester	120.00	87-19-4	0.84
12	LV_542_OKT_11	1	8.545	Butanoic acid, 2-methyl-, ethyl ester	57.00	7452-79-1	0.34
		2	9.158	1-Butanol, 3-methyl-, acetate	43.00	123-92-2	2.78
		3	9.26	3-Hexen-1-ol	41.00	928-96-1	1.07
		4	9.437	1-Hexanol	56.00	111-27-3	1.19
		5	10.177	1,2-Propanediol, 1-acetate	43.00	627-69-0	4.41
		6	10.513	1,2-Propanediol, 2-acetate	43.00	03/01/6214	2.77
		7	11.533	Hexanoic acid, ethyl ester	88.00	123-66-0	0.08
		8	11.842	Acetic acid, hexyl ester	43.00	142-92-7	0.26
		9	14.971	Butanoic acid, 3-hexenyl ester, (Z)-	67.00	16491-36-4	0.17
		10	15.144	1,2,3-Propanetriol, 1-acetate	43.00	106-61-6	6.2
		11	15.643	L-.alpha.-Terpineol	59.00	10482-56-1	0.11
		12	15.851	1,2,3-Propanetriol, 1-acetate	43.00	106-61-6	0.56
		13	16.759	Propyl 2-ethylbutanoate	43.00	5129-46-4	0.09
		14	17.769	.alpha.-d-Erythro-hex-2-enopyranoside, ethyl 2,3-dideoxy-	114.00	23339-15-3	26.94
		15	17.977	2,3-dihydroxypropyl isobutyrate	71.00	557-25-5	0.8
		16	18.285	Pyridine, 3-(1-methyl-2-pyrrolidinyl)-, (S)-	84.00	54-11-5	51.12
		17	19.19	2-Buten-1-one, 1-(2,6,6-trimethyl-1-cyclohexen-1-yl)-	177.00	23726-92-3	0.07
		18	20.311	(1′s,2′s)-Nicotine-N'-oxide	119.00	51095-86-4	0.49
		19	20.659	2(3H)-Furanone, dihydro-5-pentyl-	85.00	104-61-0	0.14
		20	20.826	Propyl 2-methylvalerate	99.00	6639-14-1	0.16
		21	24.033	Benzyl Benzoate	105.00	120-51-4	0.24
13	LV_542_OKT_12	1	8.83	3-Hexanol, 2-methyl-	55.00	617-29-8	0.08
		2	9.463	2-Propanol, 1,3-dichloro-	79.00	96-23-1	0.31
		3	9.88	2-Propenoic acid, 3-phenyl-, 2-methyl-2-propenyl ester	43.00	54889-46-2	0.15
		4	10.178	1,2-Propanediol, 1-acetate	43.00	627-69-0	0.62
		5	10.513	1,2-Propanediol, 2-acetate	43.00	03/01/6214	0.39
		6	10.812	1,2-Propanediol, 3-methoxy-	45.00	623-39-2	0.23
		7	11.552	1,3-Dioxolane, 2,2,4-trimethyl-	43.00	1193-11-9	3.19
		8	11.666	Silane, triethylmethoxy-	117.00	2117-34-2	1.04
		9	15.147	1,2,3-Propanetriol, 1-acetate	43.00	106-61-6	1.29
		10	16.077	Ethyl maltol	140.00	08/11/4940	3.2
		11	16.929	Cyclohexanol, 5-methyl-2-(1-methylethyl)-, acetate	95.00	16409-45-3	0.19
		12	17.977	2,3-dihydroxypropyl isobutyrate	71.00	557-25-5	1.15
		13	18.285	Pyridine, 3-(1-methyl-2-pyrrolidinyl)-, (S)-	84.00	54-11-5	83.77
		14	18.387	Pyridine, 3-(1-methyl-2-pyrrolidinyl)-, (S)-	84.00	54-11-5	0.57
		15	19.16	2(3H)-Furanone, dihydro-5-pentyl-	85.00	104-61-0	0.16
		16	19.753	Benzaldehyde, 3-hydroxy-4-methoxy-	152.00	621-59-0	1.54
		17	20.31	(1′s,2′s)-Nicotine-N'-oxide	119.00	51095-86-4	0.78
		18	20.523	Ethyl Vanillin	137.00	121-32-4	0.6
		19	20.659	2(3H)-Furanone, 5-heptyldihydro-	85.00	104-67-6	0.51
		20	23.392	.gamma.-Dodecalactone	85.00	07/05/2305	0.23
14	LV_542_OKT_13	1	8.756	Propylene Glycol	45.00	57-55-6	0.93
		2	9.31	Methyl propyl ether	45.00	557-17-5	0.37
		3	9.463	2-Propanol, 1,3-dichloro-	79.00	96-23-1	0.22
		4	10.176	1,2-Propanediol, 1-acetate	43.00	627-69-0	0.98
		5	10.513	1,2-Propanediol, 2-acetate	43.00	03/01/6214	0.61
		6	11.899	Methyl propionate	57.00	554-12-1	0.28
		7	14.247	Glycerin	61.00	56-81-5	0.38
		8	14.956	Isophorone	82.00	78-59-1	0.65
		9	15.145	1,2,3-Propanetriol, 1-acetate	43.00	106-61-6	1.47
		10	16.074	Ethyl maltol	140.00	08/11/4940	13.66
		11	16.604	Alpha-monopropionin	57.00	624-47-5	0.34
		12	16.907	1,3-Dioxolane, 4-methyl-2-phenyl-	163.00	2568-25-4	0.19
		13	17.193	Benzaldehyde, 4-methoxy-	135.00	123-11-5	0.3
		14	17.614	Benzenemethanol, 4-methoxy-	138.00	105-13-5	0.3
		15	17.712	5-Thiazoleethanol, 4-methyl-	112.00	137-00-8	0.3
		16	18.283	Pyridine, 3-(1-methyl-2-pyrrolidinyl)-, (S)-	84.00	54-11-5	55.85
		17	18.405	p-Dioxane-2,5-dimethanol	57.00	14236-12-5	0.29
		18	19.159	2(3H)-Furanone, dihydro-5-pentyl-	85.00	104-61-0	1.15
		19	19.752	Benzaldehyde, 3-hydroxy-4-methoxy-	151.00	621-59-0	7.09
		20	20.308	(1′s,2′s)-Nicotine-N'-oxide	119.00	51095-86-4	0.55
		21	20.519	Ethyl Vanillin	137.00	121-32-4	10.51
		22	20.637	Mandelic acid, 3,4-dimethoxy-, methyl ester	167.00	2911-73-1	3.36
		23	23.189	Vanillin propylene glycol acetal	87.00	68527-74-2	0.19
15	LV_542_OKT_14	1	8.542	Butanoic acid, 2-methyl-, ethyl ester	57.00	7452-79-1	1.14
		2	9.263	3-Hexen-1-ol, (Z)-	41.00	928-96-1	0.04
		3	9.435	1-Hexanol	56.00	111-27-3	0.32
		4	9.813	Dimethyl Sulfoxide	63.00	67-68-5	0.17
		5	10.175	1,2-Propanediol, 1-acetate	43.00	627-69-0	2.69
		6	10.51	1,2-Propanediol, 2-acetate	43.00	03/01/6214	1.77
		7	10.969	2-(2-Methoxyethoxy)ethyl acetate	43.00	629-38-9	0.05
		8	11.896	Methyl propionate	57.00	554-12-1	0.77
		9	12.046	D-Limonene	67.00	5989-27-5	0.03
		10	14.694	1-Propanol, 2-ethoxy-	45.00	19089-47-5	1.18
		11	14.858	Propanoic acid, 2-hydroxy-, methyl ester, (.±.)-	45.00	2155-30-8	0.39
		12	14.981	2-Butanol, (R)-	45.00	14898-79-4	0.35
		13	15.141	1,2,3-Propanetriol, 1-acetate	43.00	106-61-6	4.09
		14	15.847	1,2-Ethanediol, diacetate	43.00	111-55-7	0.34
		15	16.072	Ethyl maltol	140.00	08/11/4940	5.83
		16	16.6	Alpha-monopropionin	57.00	624-47-5	1.1
		17	17.766	.alpha.-d-Erythro-hex-2-enopyranoside, ethyl 2,3-dideoxy-	114.00	23339-15-3	20.92
		18	17.974	2,3-dihydroxypropyl isobutyrate	71.00	557-25-5	0.46
		19	18.283	Pyridine, 3-(1-methyl-2-pyrrolidinyl)-, (S)-	84.00	54-11-5	53.99
		20	18.374	Pyridine, 3-(1-methyl-2-pyrrolidinyl)-, (S)-	84.00	54-11-5	1.05
		21	18.467	Methyl anthranilate	119.00	134-20-3	0.33
		22	19.397	Acetic acid, (2-methoxyethoxy)-	45.00	16024-56-9	0.68
		23	19.751	Benzaldehyde, 3-hydroxy-4-methoxy-	152.00	621-59-0	0.13
		24	22.261	Cyclohexanecarboxamide, N-ethyl-5-methyl-2-(1-methylethyl)-	87.00	68489-00-9	0.72
		25	24.568	4-(4-Hydroxyphenyl)-2-butanone propyleneglycol	101.00	-	1.47
16	LV_542_OKT_15	1	8.536	Butanoic acid, 2-methyl-, ethyl ester	57.00	7452-79-1	0.17
		2	8.747	Propylene Glycol	45.00	57-55-6	0.27
		3	9.303	Methyl propyl ether	45.00	557-17-5	0.11
		4	9.456	2-Propanol, 1,3-dichloro-	79.00	96-23-1	0.32
		5	10.169	1,2-Propanediol, 1-acetate	43.00	627-69-0	2.33
		6	10.504	1,2-Propanediol, 2-acetate	43.00	03/01/6214	1.74
		7	11.483	Butanoic acid, butyl ester	71.00	109-21-7	0.05
		8	11.841	Dihydroxyacetone	42.00	96-26-4	0.19
		9	12.26	2(3H)-Furanone, dihydro-5-methyl-	56.00	108-29-2	0.1
		10	12.444	Butanoic acid, 3-methyl-, butyl ester	85.00	106-27-4	0.22
		11	15.138	1,2,3-Propanetriol, 1-acetate	43.00	106-61-6	4.76
		12	15.847	1,2-Epoxy-3-propyl acetate	43.00	-	0.54
		13	16.071	Ethyl maltol	140.00	08/11/4940	0.89
		14	18.278	Pyridine, 3-(1-methyl-2-pyrrolidinyl)-, (S)-	84.00	54-11-5	77.15
		15	18.373	Pyridine, 3-(1-methyl-2-pyrrolidinyl)-, (S)-	84.00	54-11-5	1.5
		16	18.475	Piperonal	149.00	120-57-0	1.46
		17	19.745	Benzaldehyde, 3-hydroxy-4-methoxy-	151.00	621-59-0	4.46
		18	20.304	(1′s,2′s)-Nicotine-N'-oxide	119.00	51095-86-4	0.48
		19	20.514	Ethyl Vanillin	137.00	121-32-4	1.74
		20	20.631	Benzaldehyde, 3,4-dimethoxy-, methylmonoacetal	167.00	-	1.12
		21	22.26	2H-1-Benzopyran-2-one, 6-methyl-	160.00	92-48-8	0.41
17	LV_542_OKT_16	1	8.538	Butanoic acid, 2-methyl-, ethyl ester	57.00	7452-79-1	0.76
		2	9.259	3-Hexen-1-ol, (Z)-	41.00	928-96-1	0.19
		3	9.43	1-Hexanol	56.00	111-27-3	0.33
		4	9.814	Dimethylsulfoxonium formylmethylide	63.00	-	0.11
		5	10.172	1,2-Propanediol, 1-acetate	43.00	627-69-0	0.48
		6	10.508	1,2-Propanediol, 2-acetate	43.00	03/01/6214	0.31
		7	10.653	Cyclotetrasiloxane, octamethyl-	281.00	556-67-2	0.19
		8	11.526	Hexanoic acid, ethyl ester	88.00	123-66-0	0.65
		9	11.893	Methyl propionate	57.00	554-12-1	0.26
		10	12.04	D-Limonene	68.00	5989-27-5	0.03
		11	12.631	Butanoic acid, 3-methylbutyl ester	71.00	106-27-4	0.08
		12	14.62	Maltol	126.00	118-71-8	0.54
		13	14.987	1,3-Dioxolane-2-acetic acid, 2,4-dimethyl-, ethyl ester	43.00	6290-17-1	0.17
		14	15.131	1,3-Dioxolane-2-acetic acid, 2,4-dimethyl-, ethyl ester	43.00	6290-17-1	1.07
		15	16.068	Ethyl maltol	140.00	08/11/4940	3.18
		16	16.442	Geraniol	69.00	106-24-1	0.51
		17	17.76	.alpha.-d-Erythro-hex-2-enopyranoside, ethyl 2,3-dideoxy-	114.00	23339-15-3	14.56
		18	17.97	2,3-dihydroxypropyl isobutyrate	71.00	557-25-5	0.25
		19	18.277	Pyridine, 3-(1-methyl-2-pyrrolidinyl)-, (S)-	84.00	54-11-5	68.53
		20	18.974	Cyclohexanepropanoic acid, 2-propenyl ester	55.00	2705-87-5	0.93
		21	19.474	Benzoic acid, 2-amino-, ethyl ester	119.00	87-25-2	0.09
		22	20.65	2(3H)-Furanone, 5-hexyldihydro-	85.00	706-14-9	4.83
		23	21.219	Cyclooctasiloxane, hexadecamethyl-	73.00	556-68-3	0.07
		24	22.056	2(3H)-Furanone, 5-heptyldihydro-	85.00	104-67-6	0.61
		25	23.384	.gamma.-Dodecalactone	85.00	07/05/2305	0.52
		26	24.568	4-(4-Hydroxyphenyl)-2-butanone propyleneglycol	101.00	-	0.78
18	LV_542_OKT_17	1	9.302	Methyl propyl ether	45.00	557-17-5	0.16
		2	9.456	2-Propanol, 1,3-dichloro-	79.00	96-23-1	0.09
		3	10.169	1,2-Propanediol, 1-acetate	43.00	627-69-0	0.71
		4	10.504	1,2-Propanediol, 2-acetate	43.00	03/01/6214	0.44
		5	10.652	Cyclotetrasiloxane, octamethyl-	281.00	556-67-2	0.14
		6	11.544	1,3-Dioxolane, 2,2,4-trimethyl-	43.00	1193-11-9	2.56
		7	11.659	Silane, triethylmethoxy-	117.00	2117-34-2	0.71
		8	11.775	Pyrazine, trimethyl-	42.00	14667-55-1	0.25
		9	12.582	Acetylpyrazine	43.00	22047-25-2	0.09
		10	14.617	Maltol	126.00	118-71-8	3.14
		11	15.138	1,2,3-Propanetriol, 1-acetate	43.00	106-61-6	1.84
		12	16.068	Ethyl maltol	140.00	08/11/4940	3.31
		13	17.969	2,3-dihydroxypropyl isobutyrate	71.00	557-25-5	0.34
		14	18.277	Pyridine, 3-(1-methyl-2-pyrrolidinyl)-, (S)-	84.00	54-11-5	67.54
		15	18.474	Piperonal	149.00	120-57-0	0.51
		16	19.745	Benzaldehyde, 3-hydroxy-4-methoxy-	151.00	621-59-0	11.67
		17	20.094	1,4-Benzenediol, 2-methoxy-	140.00	824-46-4	0.33
		18	20.303	(1′s,2′s)-Nicotine-N'-oxide	119.00	51095-86-4	0.59
		19	20.631	Mandelic acid, 3,4-dimethoxy-, methyl ester	167.00	2911-73-1	5.26
		20	22.305	1,3-Benzodioxole, 5-(4-methyl-1,3-dioxolan-2-yl)-	149.00	-	0.09
		21	23.182	Vanillin propylene glycol acetal	151.00	68527-74-2	0.23
19	LV_542_OKT_18	1	8.535	Butanoic acid, 2-methyl-, ethyl ester	57.00	7452-79-1	0.69
		2	8.649	2,3-Butanediol, [R-(R*,R*)]-	45.00	24347-58-8	0.08
		3	9.254	3-Hexen-1-ol, (Z)-	41.00	928-96-1	0.78
		4	9.463	2-Propanol, 1,3-dichloro-	79.00	96-23-1	0.06
		5	10.169	1,2-Propanediol, 1-acetate	43.00	627-69-0	0.43
		6	10.505	1,2-Propanediol, 2-acetate	43.00	03/01/6214	0.28
		7	11.479	Tetraethylene glycol	45.00	112-60-7	5.15
		8	11.891	Methyl propionate	57.00	554-12-1	0.95
		9	14.69	1-Propanol, 2-ethoxy-	45.00	19089-47-5	2.68
		10	14.853	2-Butanol, (R)-	45.00	14898-79-4	0.88
		11	14.976	2-Butanol, (R)-	45.00	14898-79-4	0.85
		12	15.141	1,2,3-Propanetriol, 1-acetate	43.00	106-61-6	1.11
		13	16.07	Ethyl maltol	140.00	08/11/4940	10.72
		14	16.598	Alpha-monopropionin	57.00	624-47-5	1.29
		15	17.761	.alpha.-d-Erythro-hex-2-enopyranoside, ethyl 2,3-dideoxy-	114.00	23339-15-3	4.54
		16	17.971	2,3-dihydroxypropyl isobutyrate	71.00	557-25-5	0.28
		17	18.282	Pyridine, 3-(1-methyl-2-pyrrolidinyl)-, (S)-	84.00	54-11-5	65.27
		18	18.463	Methyl anthranilate	119.00	134-20-3	0.58
		19	19.389	Acetic acid, (2-methoxyethoxy)-	45.00	16024-56-9	2.13
		20	22.258	Cyclohexanecarboxamide, N-ethyl-5-methyl-2-(1-methylethyl)-	87.00	68489-00-9	1.25
20	LV_542_OKT_19	1	8.754	Propylene Glycol	45.00	57-55-6	0.11
		2	9.457	2-Propanol, 1,3-dichloro-	79.00	96-23-1	0.33
		3	10.172	1,2-Propanediol, 1-acetate	43.00	627-69-0	23.43
		4	10.506	1,2-Propanediol, 2-acetate	43.00	03/01/6214	14.68
		5	10.653	Cyclotetrasiloxane, octamethyl-	281.00	556-67-2	0.19
		6	15.141	1,2,3-Propanetriol, 1-acetate	43.00	106-61-6	30.26
		7	15.843	1,2,3-Propanetriol, 1-acetate	43.00	106-61-6	2.99
		8	16.071	Ethyl maltol	140.00	08/11/4940	6.95
		9	17.041	1,2,3-Propanetriol, 1-acetate	43.00	106-61-6	0.12
		10	18.278	Pyridine, 3-(1-methyl-2-pyrrolidinyl)-, (S)-	84.00	54-11-5	16.61
		11	19.156	2(3H)-Furanone, dihydro-5-pentyl-	85.00	104-61-0	0.21
		12	19.748	Benzaldehyde, 3-hydroxy-4-methoxy-	151.00	621-59-0	2.96
		13	20.306	(1′s,2′s)-Nicotine-N'-oxide	119.00	51095-86-4	0.21
		14	20.518	Ethyl Vanillin	137.00	121-32-4	0.14
		15	20.634	Mandelic acid, 3,4-dimethoxy-, methyl ester	167.00	2911-73-1	0.8
21	LV_542_OKT_20	1	8.613	Butanoic acid, 2-methyl-, ethyl ester	57.00	7452-79-1	2.26
		2	9.352	3-Hexen-1-ol, (Z)-	41.00	928-96-1	8.91
		3	9.521	1-Hexanol	56.00	111-27-3	1.49
		4	9.643	Butanoic acid, 2-methyl-	74.00	116-53-0	1.96
		5	12.152	Hexanoic acid	60.00	142-62-1	1.72
		6	16.199	Ethyl maltol	140.00	08/11/4940	4.43
		7	18.419	Pyridine, 3-(1-methyl-2-pyrrolidinyl)-, (S)-	84.00	54-11-5	71.86
		8	18.905	2-Propenoic acid, 3-phenyl-, methyl ester	131.00	1754-62-7	2.05
		9	20.809	2(3H)-Furanone, 5-hexyldihydro-	85.00	706-14-9	4.58
		10	23.98	.delta.-Dodecalactone	99.00	713-95-1	0.73
22	LV_542_OKT_21	1	9.11	Furfural	96.00	98-01-1	0.57
		2	9.69	2-Furanmethanol	98.00	98-00-0	0.06
		3	10.262	1,2-Propanediol, 1-acetate	43.00	627-69-0	3.21
		4	10.599	1,2-Propanediol, 2-acetate	43.00	03/01/6214	1.84
		5	12.507	1,2-Propanediol, diacetate	43.00	623-84-7	0.15
		6	14.738	Maltol	126.00	118-71-8	5.68
		7	15.267	1,2,3-Propanetriol, 1-acetate	43.00	106-61-6	2.75
		8	15.689	2-Acetoxyisobutyryl chloride	43.00	40635-66-3	0.37
		9	15.874	2-Acetoxyisobutyryl chloride	43.00	40635-66-3	1.03
		10	16.074	2-Acetoxyisobutyryl chloride	43.00	40635-66-3	2.76
		11	16.197	Ethyl maltol	140.00	08/11/4940	6.86
		12	16.635	4-Methyl-2-oxopentanenitrile	43.00	66582-16-9	0.17
		13	17.319	Benzaldehyde, 4-methoxy-	135.00	123-11-5	0.16
		14	18.115	2,3-dihydroxypropyl isobutyrate	71.00	557-25-5	0.55
		15	18.419	Pyridine, 3-(1-methyl-2-pyrrolidinyl)-, (S)-	84.00	54-11-5	67.33
		16	18.618	Piperonal	149.00	120-57-0	0.53
		17	19.3	2(3H)-Furanone, dihydro-5-pentyl-	85.00	104-61-0	0.48
		18	19.897	Vanillin	152.00	121-33-5	2.16
		19	20.458	(1′s,2′s)-Nicotine-N'-oxide	60.00	51095-86-4	0.2
		20	20.671	Ethyl Vanillin	137.00	121-32-4	2.31
		21	21.238	1,2,3,6-Tetrahydro-2,3′-bipyridine	54.00	2743-90-0	0.11
		22	23.981	.delta.-Dodecalactone	99.00	713-95-1	0.39
		23	27.464	n-Nonadecanol-1	55.00	1454-84-8	0.14
		24	27.803	Methyl stearate	74.00	112-61-8	0.19
23	LV_542_OKT_22	1	9.232	1-Butanol, 3-methyl-, acetate	43.00	123-92-2	1.05
		2	9.348	3-Hexen-1-ol, (Z)-	41.00	928-96-1	3.01
		3	9.52	1-Hexanol	56.00	111-27-3	0.53
		4	9.654	Butanoic acid, 2-methyl-	74.00	116-53-0	0.09
		5	10.264	1,2-Propanediol, 1-acetate	43.00	627-69-0	0.71
		6	10.6	1,2-Propanediol, 2-acetate	43.00	03/01/6214	0.44
		7	11.621	Hexanoic acid, ethyl ester	88.00	123-66-0	0.04
		8	11.684	Benzaldehyde	77.00	100-52-7	0.08
		9	11.768	1-Dimethyl(octyl)silyloxypropane	117.00	-	0.41
		10	11.931	Acetic acid, hexyl ester	43.00	142-92-7	0.42
		11	15.266	1,2,3-Propanetriol, 1-acetate	43.00	106-61-6	1.11
		12	15.553	Benzenemethanol, .alpha.-methyl-, acetate	122.00	93-92-5	0.45
		13	16.016	Benzoic acid	105.00	65-85-0	1.34
		14	16.198	Ethyl maltol	140.00	08/11/4940	1.19
		15	16.573	Geraniol	69.00	106-24-1	1.06
		16	17.033	1,3-Dioxolane, 4-methyl-2-phenyl-	105.00	2568-25-4	0.07
		17	17.594	Dianhydromannitol	86.00	19895-66-0	0.24
		18	17.704	2(3H)-Furanone, 5-butyldihydro-	85.00	104-50-7	0.99
		19	17.902	.alpha.-d-Erythro-hex-2-enopyranoside, ethyl 2,3-dideoxy-	114.00	23339-15-3	23.15
		20	18.232	1,4-Dioxane-2,6-dimethanol	57.00	54120-69-3	0.29
		21	18.422	Pyridine, 3-(1-methyl-2-pyrrolidinyl)-, (S)-	84.00	54-11-5	57.26
		22	18.556	p-Dioxane-2,5-dimethanol	57.00	14236-12-5	0.36
		23	19.122	Cyclohexanepropanoic acid, 2-propenyl ester	55.00	2705-87-5	0.11
		24	20.459	(1′s,2′s)-Nicotine-N'-oxide	60.00	51095-86-4	0.19
		25	20.81	2(3H)-Furanone, 5-hexyldihydro-	85.00	706-14-9	5.32
		26	27.462	1-Octadecanol	57.00	112-92-5	0.05
24	LV_542_OKT_23	1	8.626	Butanoic acid, 2-methyl-, ethyl ester	57.00	7452-79-1	0.49
		2	9.239	1-Butanol, 3-methyl-, acetate	43.00	123-92-2	0.17
		3	9.518	1-Hexanol	56.00	111-27-3	0.11
		4	10.262	1,2-Propanediol, 1-acetate	43.00	627-69-0	0.15
		5	10.599	1,2-Propanediol, 2-acetate	43.00	03/01/6214	0.08
		6	11.574	Butanoic acid, butyl ester	71.00	109-21-7	0.02
		7	11.933	Acetic acid, hexyl ester	43.00	142-92-7	0.12
		8	11.99	Methyl propionate	57.00	554-12-1	0.08
		9	12.07	Glycolaldehyde dimethyl acetal	75.00	30934-97-5	0.03
		10	12.134	D-Limonene	68.00	5989-27-5	0.02
		11	15.279	1,2,3-Propanetriol, 1-acetate	43.00	106-61-6	0.71
		12	16.073	Benzoic acid	105.00	65-85-0	11.35
		13	16.199	Ethyl maltol	140.00	08/11/4940	1.05
		14	16.637	4-Methyl-2-oxopentanenitrile	43.00	66582-16-9	0.05
		15	16.74	Alpha-monopropionin	57.00	624-47-5	0.29
		16	17.609	Isosorbide	86.00	652-67-5	0.14
		17	17.916	.alpha.-d-Erythro-hex-2-enopyranoside, ethyl 2,3-dideoxy-	114.00	23339-15-3	33.42
		18	18.431	Pyridine, 3-(1-methyl-2-pyrrolidinyl)-, (S)-	84.00	54-11-5	51.08
		19	18.606	Methyl anthranilate	119.00	134-20-3	0.45
		20	22.426	Cyclohexanecarboxamide, N-ethyl-5-methyl-2-(1-methylethyl)-	87.00	68489-00-9	0.19
25	LV_542_OKT_24	1	8.61	Butanoic acid, 2-methyl-, ethyl ester	57.00	7452-79-1	1.49
		2	9.346	3-Hexen-1-ol, (Z)-	41.00	928-96-1	0.65
		3	9.59	2-Propanol, 1,3-dichloro-	79.00	96-23-1	0.08
		4	9.651	Butanoic acid, 2-methyl-	74.00	116-53-0	0.06
		5	10.262	1,2-Propanediol, 1-acetate	43.00	627-69-0	0.35
		6	10.598	1,2-Propanediol, 2-acetate	43.00	03/01/6214	0.23
		7	11.202	Decane	43.00	124-18-5	0.01
		8	11.57	Butanoic acid, butyl ester	71.00	109-21-7	0.03
		9	11.849	3-Hexen-1-ol, acetate, (Z)-	43.00	3681-71-8	0.09
		10	14.731	Maltol	126.00	118-71-8	1.18
		11	16.04	Benzoic acid	105.00	65-85-0	9.56
		12	18.42	Pyridine, 3-(1-methyl-2-pyrrolidinyl)-, (S)-	84.00	54-11-5	85.83
		13	20.451	(1′s,2′s)-Nicotine-N'-oxide	119.00	51095-86-4	0.13
		14	20.802	2(3H)-Furanone, 5-hexyldihydro-	85.00	706-14-9	0.19
		15	22.217	2(3H)-Furanone, 5-heptyldihydro-	85.00	104-67-6	0.13
26	LV_542_OKT_25	1	8.617	Butanoic acid, 2-methyl-, ethyl ester	57.00	7452-79-1	0.38
		2	8.836	Propylene Glycol	45.00	57-55-6	0.16
		3	9.341	3-Hexen-1-ol, (Z)-	41.00	928-96-1	0.39
		4	9.573	Propanoic acid, chloro-2-hydroxy-	79.00	-	0.03
		5	10.254	1,2-Propanediol, 1-acetate	43.00	627-69-0	0.48
		6	10.591	1,2-Propanediol, 2-acetate	43.00	03/01/6214	0.3
		7	11.632	1,3-Dioxolane, 2,2,4-trimethyl-	43.00	1193-11-9	0.73
		8	11.865	Pyrazine, trimethyl-	42.00	14667-55-1	0.19
		9	12.676	Acetylpyrazine	43.00	22047-25-2	0.06
		10	14.729	Maltol	126.00	118-71-8	1.66
		11	15.263	1,2,3-Propanetriol, 1-acetate	43.00	106-61-6	1.54
		12	16.063	Benzoic acid	105.00	65-85-0	16.19
		13	16.188	Ethyl maltol	140.00	08/11/4940	1.43
		14	18.422	Pyridine, 3-(1-methyl-2-pyrrolidinyl)-, (S)-	84.00	54-11-5	71.04
		15	19.891	Vanillin	151.00	121-33-5	5.01
		16	20.451	(1′s,2′s)-Nicotine-N'-oxide	119.00	51095-86-4	0.31
		17	23.349	Vanillin propylene glycol acetal	87.00	68527-74-2	0.15
27	NIC_IPIN_MAY	1	8.677	Formamide, N-methyl-	59.00	123-39-7	0.6
		2	8.89	Ethylbenzene	91.00	100-41-4	0.09
		3	11.645	Octane, 3,3-dimethyl-	71.00	4110-44-5	0.04
		4	19.872	Pyridine, 3-(3,4-dihydro-2H-pyrrol-5-yl)-	118.00	532-12-7	34.36
		5	20.752	Anabasine	84.00	13078-04-1	4.96
		6	21.211	1,2,3,6-Tetrahydro-2,3′-bipyridine	54.00	2743-90-0	5.49
		7	24.447	Cotinine	98.00	486-56-6	54.46

1 Retention time

The GC-MS results for e-liquid samples, identifying various compounds and their relative abundance. Among the most frequently detected compounds are nicotine, glycerol and propylene glycol derivatives, and flavouring agents. The most common compound, Pyridine, 3-(1-methyl-2-pyrrolidinyl)-, (S)-, is a nicotine form appearing in all samples except LV_542_OKT_5B. Other notable compounds include 1,2,3-Propanetriol, 1-acetate (Triacetin), 1,2-Propanediol acetates (propylene glycol derivatives), Ethyl maltol, and 2-Propanol, 1,3-dichloro-. These substances serve as carriers, solvents, and flavouring agents in e-liquids. However, their safety upon inhalation remains a subject of ongoing research.

Glycerol and propylene glycol derivatives such as Triacetin and 1,2-Propanediol acetates are commonly used as solvents and carriers in e-liquids. While they are deemed safe for ingestion, their inhalation safety is less certain. Studies indicate that propylene glycol can cause respiratory irritation. When heated, propylene glycol can generate harmful byproducts like acrolein and formaldehyde [14], which are toxic and potentially carcinogenic.

Flavouring agents such as ethyl maltol are widely used in e-liquids to enhance sweetness and aroma. However, recent research suggests that when heated, ethyl maltol can contribute to increased free radical production in e-cigarette aerosols, potentially amplifying oxidative stress and toxicity. Additionally, certain buttery or creamy flavouring agents, such as diacetyl and acetylpropionyl, have been linked to bronchiolitis obliterans (“popcorn lung”), a severe and irreversible lung disease. The detection of 2-Propanol, 1,3-dichloro-, an industrial solvent, raises potential concerns regarding respiratory tract irritation and long-term health effects. Although data on its inhalation toxicity are limited, chemically similar compounds have been linked to adverse respiratory outcomes.

LV_542_OKT_5B, known as a booster on packaging, contain 99.85% Hexanamide, 3,5,5-trimethyl-N-pentyl-. Hexanamide, 3,5,5-trimethyl-N-pentyl- is an organic compound belonging to the amide family, which is characterised by the presence of a carbonyl group (C=O) attached to a nitrogen atom. This compound has a long-chain hydrocarbon structure, making it relatively non-polar and likely contributing to its low volatility and potential stability in various formulations. It is commonly served as a flavouring additive designed to enhance the sensory experience of the vapour such as creamy, nutty, or slightly savoury tastes. Amides are sometimes used to create a smooth, velvety mouthfeel, potentially reducing the harshness of nicotine or other intense flavour compounds. Additionally, it may serve as a binding agent, stabilizing other flavour molecules and prolonging the overall taste experience.

NIC_IPIN_MAY is obviously containing derivatives of nicotine which is dominate by Cotinine (54.455%), followed by Pyridine, 3-(3,4-dihydro-2H-pyrrol-5-yl)- (34.357%), Anabasine (4.959%) and 1,2,3,6-Tetrahydro-2,3′-bipyridine (5.494%) [15,16]. The common names for Pyridine, 3-(3,4-dihydro-2H-pyrrol-5-yl)- and 1,2,3,6-Tetrahydro-2,3′-bipyridine are myosmine and anatabine, respectively. Nicotine and its derivatives are well-known for their effects on the central nervous system, causing increased heart rate, elevated blood pressure, and strong addictive properties. Nicotine-N'-oxide, derived from nicotine, is present in 13 samples. Generally, it is considered less potent than nicotine and its presence suggests oxidation reactions in e-liquid formulations. Long-term exposure to nicotine has been linked to cardiovascular diseases and other health risks.

These data provide a foundation for further quantitative analyses and comparative studies. Further research is recommended to confirm the identities of key compounds through standard comparisons and to investigate the potential sources and implications of the identified substances.

## 4 Experimental Design, Materials and Methods

### 4.1 FTIR analysis

The samples were stored in a dry, room-temperature environment before analysis. The FTIR spectra were acquired using a Bruker INVENIO-R (Universiti Putra Malaysia) spectrometer equipped with an attenuated total reflection (ATR) (2mm) diamond. The spectra acquisition involved 64 scans with a spectral resolution of 4 cm^−1^ between 80 and 6000 cm^−1^. With a temperature control unit, the temperature is maintained at room temperature ∼26 °C during the spectra acquisition. Before the IR spectra of the sample were acquired, acetone was wiped on the ATR to remove contaminants from the previous sample which evaporated to dryness. The background spectrum is collected, which will subtract any unwanted residual peaks from the sample spectrum and avoid the contaminants reading. Then, the sample spectra were recorded immediately on the ATR and analysed using OPUS 8.7.

The IR spectra were processed using the standard peak-picking method with a sensitivity of 1.0%. The analysis focused on the nicotine characteristic peak at 714 cm^−1^, corresponding to the out of plane C-H bending of the monosubstituted pyridinic ring [12]. Upon visual inspection, other characteristic IR peaks of nicotine in the e-liquid samples were not observed very likely due to the signal overlapping with ingredients in the sample composition, including solvents (which are propylene glycol, vegetable glycerine), flavourings, sweeteners, and colourings.

### 4.2 NMR sample preparation & analysis

200 µl of each e-liquid withdrawn with a glass precision syringe were diluted with 400 µl of MeOD and mixed with 30 µl of a TSP solution (10 mM in MeOD) as reference for ^1^H NMR experiments. A similar protocol was used for sample preparation in DMSO-d_6_. All samples of e-liquids were analysed in the two solvents.

1D ^1^H NMR experiments were acquired at 298 K. Acquisition parameters were as follows: acquisition time of 4.58 s with 64 K data points, relaxation delay of 2 s, number of scans of 32 and spectral width of 12 ppm; the recording time was thus ca. 3.5 min. Data were processed with one level of zero-filling and Fourier transformation after multiplying FIDs by an exponential line-broadening function of 0.3 Hz. Phase adjustment and polynomial baseline correction were done manually on each spectrum. The signal of TSP set at 0 ppm was used as an internal reference for chemical shift measurement.

### 4.3 GC-MS sample preparation & analysis

Each e-liquid sample was homogenised using a shaker at 1800 rpm for 1 min to ensure uniformity. Subsequently, 10 µL of the e-liquid sample was transferred into a 2 mL glass GC vial and diluted with 1000 µL of methanol. The mixture was homogenised again for 1 minute before GC-MS analysis.

GC-MS analyses were conducted using a Shimadzu Nexis GC-2030 gas chromatograph coupled with a GC-MS-QP2020 NX single quadrupole mass spectrometer, equipped with an AOC-20s auto-sampler and AOC-20i auto-injector.

• The injector temperature was set to 280°C.
• A splitless injection mode was applied for 1 min, with an injection volume of 0.5 µL.
• The analytes were separated using an SH-I-624Sil MS column (30 m × 0.32 mm, 1.8 µm film thickness) with medium polarity.
• Helium was used as the carrier gas at a constant flow rate of 1.8 mL/min under constant-pressure mode.

The ion source and transfer line temperatures of the mass spectrometer were set at 230°C and 280°C, respectively. The total GC-MS run time was 35 min, with detection beginning at 8.5 min after the solvent cut time.

From 13-14 min, the detector was temporarily closed to prevent signal saturation caused by the high-intensity glycerine (e-liquid solvent) peak. This step was necessary as glycerine produces a large chromatographic peak, which can obscure low-intensity peaks of the target analytes.

The LabSolutions GC-MS software was utilized for instrument control, data acquisition, and processing. Compound identification was performed by comparing the retention times and mass spectra of detected peaks with reference standards. The NIST 17 Mass Spectral Library served as the primary reference for spectral matching.

## Limitations

Not applicable.

## Ethics Statement

The work does not involve human subjects, animal experiments, or data collected from social media platforms.

## Credit Author Statement

**Mohd Rashidi Abdull Manap:** Conceptualisation, Funding, Methodology, Writing– original draft, Supervision; **Nur Hayatna Mukhni:** Methodology, Validation, Visualisation, Writing – original draft; **Farah Natasha Mohd Aris:** Writing – analysis; **Noor Hazfalinda Hamzah:** Editing, Funding, Review, Resources, Investigation; **Saïda Danoun:** Investigation; **Stéphane Balayssac:** Investigation, Visualization, Methodology, Writing - original draft; **Véronique Gilard:** Methodology, Visualization, Writing - original draft.

## Data Availability

Mendeley DataThe MID spectra of e liquids were acquired using a Bruker Alpha II (Universiti Putra Malaysia) instrument equipped with an attenuated total internal reflection (ATR) (Original data). Mendeley DataThe FIR MID NIR spectra of e liquids were acquired using a Bruker Invenio-R (Universiti Putra Malaysia) instrument equipped with an attenuated total internal reflection (ATR) (Original data). Mendeley DataThe GCMS Chromatogram of E-liquids (Original data). Mendeley DataThe 1D 1H NMR spectra of e-liquids (Original data).
